# JNK signaling and integrins cooperate to maintain cell adhesion during epithelial fusion in *Drosophila*

**DOI:** 10.3389/fcell.2023.1034484

**Published:** 2024-01-09

**Authors:** Katerina Karkali, Jose Carlos Pastor-Pareja, Enrique Martin-Blanco

**Affiliations:** ^1^ Instituto de Biologia Molecular de Barcelona, Consejo Superior de Investigaciones Científicas (IBMB-CSIC), Barcelona, Spain; ^2^ Instituto de Neurociencias, Consejo Superior de Investigaciones Científicas (IN-CSIC), Alicante, Spain

**Keywords:** *Drosophila*, JNK, puckered, integrin, dorsal closure, JNK sensor

## Abstract

The fusion of epithelial sheets is an essential and conserved morphogenetic event that requires the maintenance of tissue continuity. This is secured by membrane-bound or diffusible signals that instruct the epithelial cells, in a coordinated fashion, to change shapes and adhesive properties and when, how and where to move. Here we show that during Dorsal Closure (DC) in *Drosophila,* the Jun kinase (JNK) signaling pathway modulates integrins expression and ensures tissue endurance. An excess of JNK activity, as an outcome of a failure in the negative feedback implemented by the dual-specificity phosphatase Puckered (Puc), promotes the loss of integrins [the ß-subunit Myospheroid (Mys)] and amnioserosa detachment. Likewise, integrins signal back to the pathway to regulate the duration and strength of JNK activity. Mys is necessary for the regulation of JNK activity levels and in its absence, *puc* expression is downregulated and JNK activity increases.

## Introduction

A wide range of morphogenetic movements, including cell delamination and condensations, and cell sheet invaginations, folding, spreading and fusions, are necessary for the completion of the developmental programs that direct the final form of tissues and organisms. During development, in many morphogenetic instances, epithelia fuse to isolate the internal tissues from the external environment. The maintenance of the continuity of epithelial layers is fundamental for life viability. A highly dynamic regulatory control of cell-to-cell and cell-to matrix adhesion appears to be essential for the completion of epithelial fusion. Two classes of molecules cooperate closely to achieve this goal, signaling pathways elements such as receptors, secondary messengers, members of phosphorylation cascades and transcription factors, and adhesion proteins such as matrix receptors (integrins), matrix-degrading enzymes and homophilic adhesion molecules, such as cadherins.

The *Drosophila* embryonic dorsal closure (DC) has become a standard model system for the study of the regulatory and cellular mechanics involved in cell sheet morphogenesis (Knust, 1997; Martín-Blanco, 1997). Equivalent processes could be found in many other developmental models; e.g., ventral enclosure in *Caenorhabditis elegans* (Raich et al., 1999), epiboly, eyelid fusion, and secondary palate fusion in vertebrates (Ferguson, 1994; Carroll et al., 1998); as well as during epidermal wound healing, both in invertebrate and vertebrate systems (Martin, 1997). During DC, the lateral epithelia on each side of the embryo undergo coordinated cell shape changes and move dorsally leading to the matching of contralateral segments at the dorsal midline. The completion of closure internalizes the amnioserosa (AS), an extraembryonic monolayered epithelium, and seals the embryo allowing successful larval hatching (Martinez Arias, 1993). The AS and epidermis communicate back and forth at several steps during the process employing mechanical and chemical signals and both contribute in different degrees to closure (Lada et al., 2012). First, it has been proposed that, during DC, a supracellular contractile purse-string, positioned at the leading edge (LE) of the lateral epidermis, directs the shape changes of both AS and epidermal cells (Young et al., 1993). This cable formation is dependent on the planar polarization of the tissue. The dorsal most epidermal cells, which lie at the LE and therefore abuts the AS, elongate within the dorsal–ventral (DV) plane. This polarity is reflected at the molecular level by the planar polarized localization of several factors, such as actin regulators that become enriched at tricellular junctions and septate junction (SJ) proteins that are absent from the front of the LE (Narasimha et al., 2008). Planar polarization is dependent on the asymmetric distribution of the homophilic cell adhesion molecule Echinoid (Ed), which disappears from the AS just before dorsal closure (Laplante and Nilson, 2011). Notably, the actomyosin cable does not seem to be the major contributor to the dynamics of closure. Rather, it shield the structure of the LE from interference by adjacent mechanical tensions, as, e.g., those concomitant to segmental grooves morphogenesis (Ducuing and Vincent, 2016). Second, beyond the actomyosin cable, dynamic filopodia and lamellipodia extending from LE cells at the final stage of closure interdigitating with filopodia from the opposing epidermal front are key for the active zipping of the epidermal sheets (Millard and Martin, 2008). Further, pulsatile non-muscle myosin II-driven constriction in the AS (Solon et al., 2009; Blanchard et al., 2010), AS cells’ death (Toyama et al., 2008) and lateral epidermis counteracting forces (Lv et al., 2022) are essential driving elements for the process.

How the intercellular and mechanical interactions between the LE, epidermal and AS cells and underlying yolk cell are implemented is not fully understood. Jun kinase (JNK—Basket (Bsk) in *Drosophila*) signaling has been shown to play a key role in regulating DC (Glise et al., 1995; Zeitlinger et al., 1997). The JNK signaling cascade is initially active in both the AS and the LE, and is downregulated in the AS, but not in the LE, prior to closure (Reed et al., 2001). These LE cells constitute the signaling center responsible for coordinating the whole process. The most upstream acting molecules identified for JNK signaling cascade activation during DC are non-receptor tyrosine kinases of the Src family: Src42A, Src64B, Btk29A and Shark. Further, Rho family G proteins, Rac (Rac1, Rac2, and Mtl) and Cdc42, become involved [Reviewed in (Rios-Barrera and Riesgo-Escovar, 2013)]. Downstream, the JNK cascade activates the AP-1 transcription factor comprised of DJun and DFos that, in turn, upregulates the expression of the *decapentaplegic (dpp)* and *puckered (puc)* genes (Glise and Noselli, 1997; Hou et al., 1997; Riesgo-Escovar and Hafen, 1997). Dpp is a secreted morphogen that signals lateral epidermal cells to elongate along the dorsoventral axis (Affolter et al., 1994). *puc* encodes a phosphatase that negatively regulates the kinase activity of JNK (Martín-Blanco et al., 1998). This negative feedback provides one mechanism with which to limit the level of signaling through the JNK pathway. JNK signaling also activates expression of Chickadee, the fly homolog of vertebrate profilins, a well-known regulator of the actin cytoskeleton (Jasper et al., 2001). Other JNK targets include matrix metalloproteinases, stress-related proteins and, importantly, integrins (Wang et al., 2003; Homsy et al., 2006; Stevens and Page-McCaw, 2012). Integrins are mediators of cell-cell contacts and cell-matrix interactions essentials to hold AS integrity and AS/LE attachments. Strong expression of integrins is found along the entire AS/LE interface, partially colocalizing with laminin, a basement membrane integrin ligand (Reed et al., 2004). At these cell-cell contacts, integrins are also found at apical Adherens Junctions (AJs) (Narasimha and Brown, 2004). Further, integrins are present at the basal surface of the AS implementing contacts to the yolk cell membrane. This adhesion is essential for AS integrity and its contractile activity (Narasimha and Brown, 2004; Reed et al., 2004). In mutants lacking the integrin βPS subunit the AS detaches from the underlying yolk cell (Homsy et al., 2006; Peralta et al., 2007; Wada et al., 2007). The weakening of the AS/LE interface will lead to its disassembly. The AS continues autonomously contracting unrestricted from the resistance generated by the epidermis providing *in vivo* evidence for the coordination of inter tissue mechanics during DC (Gorfinkiel et al., 2009). As integrins can transduce traction forces generated by actomyosin networks (Hu et al., 2007), it is possible that during DC they may play a similar role (Goodwin et al., 2016).

A balance of forces between the AS and the epidermis in response to JNK activity at the LE mediated by integrins might be essential to lead efficient DC. Further, defects triggered by the loss of attachment at the AS/LE interface might amplify and be amplified by weaker yolk cell-AS contacts. To investigate how the JNK signaling cascade affects cell behaviors and mechanics during DC, we have imaged wild type and JNK signaling mutant embryos at high resolution monitoring newly developed JNK activity biosensors. Interestingly, we observed in that, in contrast to the expected downregulation of JNK activity at the LE associated to the detachment of epidermal and AS cells, this is sustained, although it becomes irregularly distributed. We found that optimal JNK signaling levels were necessary to maintain the correct expression of integrins at the AS/LE interface and the AS as previously described and uncovered a positive reinforcement of JNK signaling activity by integrins at the LE. Both integrins and JNK signaling are necessary to sustain the planar polarity of LE cells during DC progression.

## Results

### JNK signaling is necessary for maintaining the adhesion between epidermal and AS cells during DC

**FIGURE 1:**
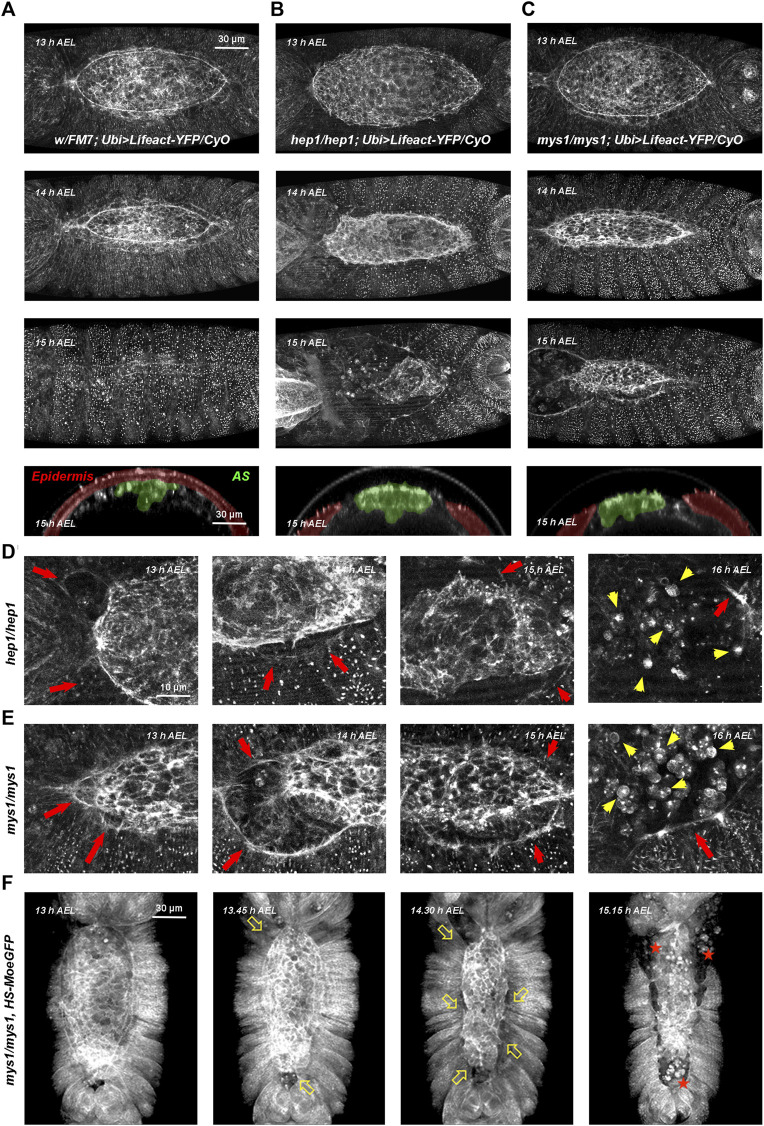
Wild type, *hep* and *mys* mutant embryos through DC **(A–C)** Embryos with dorsal orientations give an overall view of the adhesion defects observed in wild type and mutant embryos during DC at different times [13–15 h after egg laying (AEL)] and cross-sections at 15 h AEL (Ubi-Lifeact-YFP—see Materials and Methods). **(A)** Wild type embryos. At 13 h AEL, DC is 40% completed; at 14 h, DC is 75% completed and at 15 h DC is finished. Cross-section at DC completion show the internalized AS cells (pseudo colored in green) and the sealed embryonic epidermis (pseudo colored in magenta). **(B)** *hep1* mutant embryos. The actin cable gets progressively disassembles and the AS detaches and disaggregates. **(C)** *mys1* mutant embryos. The AS detaches, but the actin cable at the LE is sustained. For both, *hep1* and *mys1* mutants, cross-sections at late stages show the detachment of the AS from the LE. See Supplementary Movies S1, S3, S5. **(D, E)** High magnification images of the actin cytoskeleton of *hep1* **(D)** and *mys1* mutants **(E)** monitored by the expression of Lifeact-YFP along DC progression. Magenta arrows point to the LE, detached from the AS. Note the maintenance of the LE actin cable. Yellow arrows point to the disassembled cells of the AS undergoing rounding and apoptosis. **(F)** Low magnification snapshots from video time-lapse images of a *mys* ^*1*^ mutant embryo expressing Moesin-GFP fusion protein (HS-Moe-GFP). The epidermis detaches from the AS and initiates retraction (arrows). AS cells undergo anoikis (stars). Dorsal views, anterior is up. Scale bars are as indicated in the different panels.

Mutants for different components of the JNK signaling pathway fail to accumulate F-actin and non-muscle myosin II, and to assemble filopodia at the LE during DC (Young et al., 1993; Jacinto et al., 2000). It has also been shown that the elongation of the lateral epidermis is impaired in these conditions. In order to define more precisely the cellular effects of interfering with JNK signaling we performed an *in vivo* analysis of live embryos expressing two different markers, LifeAct-YFP (Figure 1) (Brock et al., 2012) or Moesin-GFP (Supplementary Figure S1) (Edwards et al., 1997), monitoring both cell integrity and actin cytoskeleton dynamics.

In wild type embryos, for both markers, a thick actin cable is observed at the LE. The epidermal cells coordinately stretch concurrently with the constriction of the very flat AS cells, which sink into the interior of the embryo. The cells of the lateral epidermis do not advance over the cells of the AS, as previously shown (Rugendorff et al., 1994). Upon closure, the epidermis secretes the larval cuticle and no scar is left (see Figure 1A and Supplementary Figures S1A–D and Supplementary Movies S1, S2).

In embryos deficient for JNK signaling, in the absence of JNKK, the JNK kinase encoded by the gene *hemipterous* (*hep*), filamentous actin levels were strongly reduced at the LE and both, the epidermis and the AS retracted autonomously (Figures 1B, D, red arrows). AS cells detached from each other and the LE and died in a process previously described as a matrix detachment-induced apoptosis (anoikis) (Frisch and Screaton, 2001; Reed et al., 2004) (Figure 1D, yellow arrows). The gut and other internal structures were eventually extruded from the interior of the embryo (see also Supplementary Movies S3, S4).

In summary, our observations suggest that the primary defect in *hep* mutants consist of the loss of adhesion between the AS and the lateral epidermis at the LE. This does not preclude other possible direct or indirect roles of JNK signaling in epidermal cell elongation and polarization, or in the plasticity of AS cells.

### Cell adhesion during DC depends on myospheroid expression and this is controlled by JNK activity

In light of the adhesion defects observed in *hep* mutants, we aimed to search for adhesion molecules that would eventually hold together the AS and the lateral epidermis. It was previously shown that altering JNK activity in the AS or the lateral epidermis leads to defective focal complexes (phosphotyrosine staining) assembled at the boundary LE cells (Harden et al., 1995; Reed et al., 2001). One hypothesis for a potential mechanism for adhesion is that components of focal complexes may be sensitive to JNK signaling and that alterations in these components would affect the attachment between the AS and the LE cells.

The major transmembrane receptors in focal complexes are the integrins. Integrins are a family of transmembrane heterodimeric proteins (α and ß subunits) that mediate cell-extracellular matrix and cell-cell adhesive interactions and participate in signaling across the plasma membrane (Keely et al., 1998). The exact subunit combination dictates the binding specificity of the integrin to different ECM components. They play critical roles in adhesion, migration, morphogenesis, and the differentiation of several cell types. Their levels and activities are modulated in a wide variety of biological processes. Phenotypes of *myospheroid* (*mys*)*,* a *Drosophila* ß-subunit coding gene, mutant embryos include: separation and twisting of the embryonic germ band, detachment of somatic muscles, abnormal shape and migration of midgut primordia, and detachment of AS cells and rupture of the embryonic cuticle along the dorsal midline (Wright, 1960). Time-lapse video microscopy of LifeAct-YFP (Figures 1C, E and Supplementary Movie S5) and Moesin-GFP-expressing *mys* mutant embryos (Figure 1F), show premature detachment of AS and LE cells in an equivalent way to *hep* mutant embryos. The AS and the lateral epidermis tear away from each other and AS cells undergo apoptosis (Supplementary Figure S3).

Then, we studied in detail, dynamically, the effect of reducing integrins expression. In the ectoderm of wild type embryos, in accord with previous reports (Narasimha and Brown, 2004; Reed et al., 2004), Mys is expressed at the surface of AS cells and at the leading front of LE cells during DC (Supplementary Figure S2A). We applied super resolution microscopy and found, by evaluating colocalization of AS and epidermal markers, that Mys is expressed in a double row at the AS/LE interface at Adherens Junctions (Supplementary Figures S2B, C).

**FIGURE 2:**
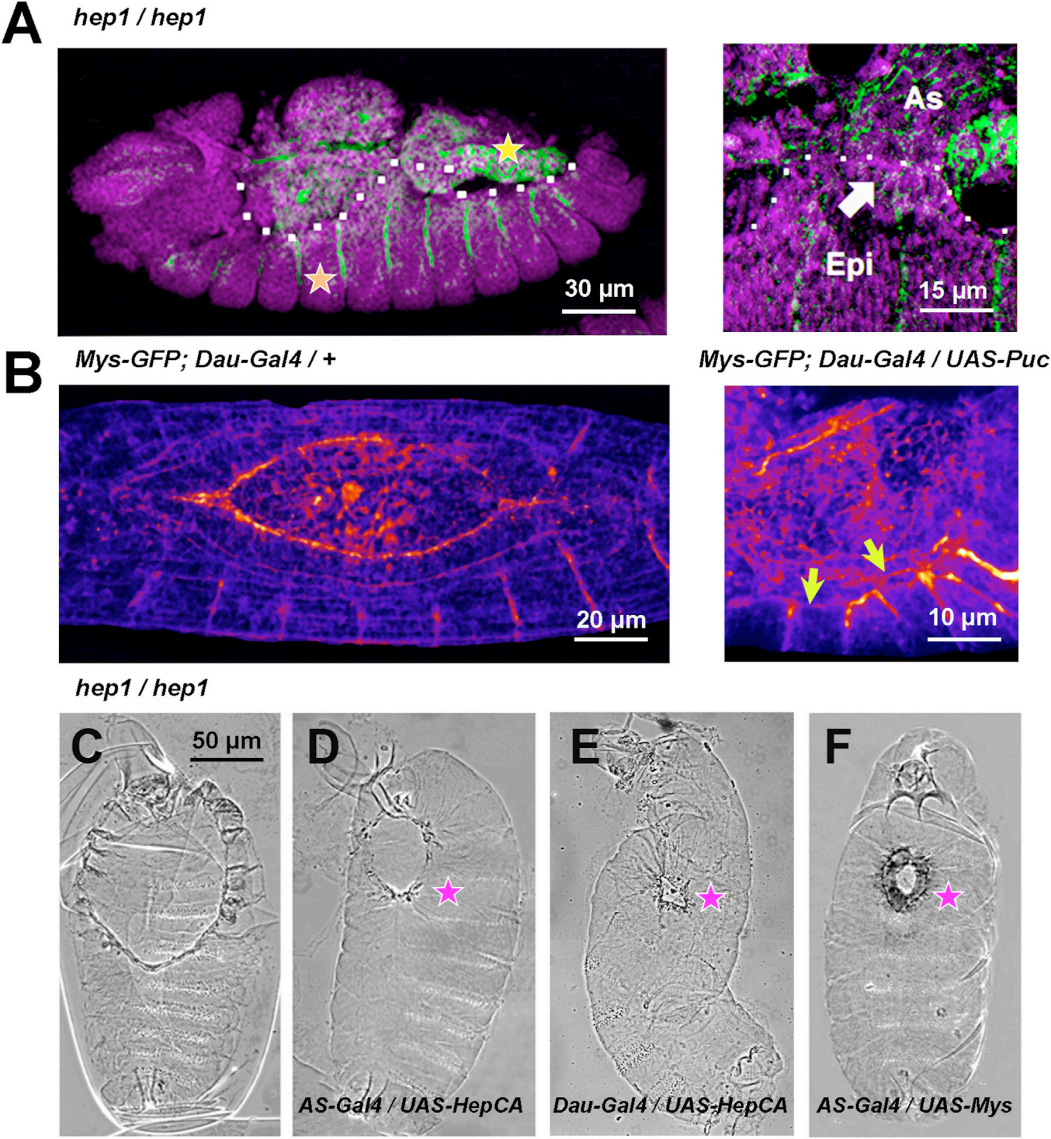
JNK signaling modulates Mys expression and AS/LE attachment **(A)** *hep* ^*1*^ mutant embryos. Mys expression (green) is lost from the LE cells (arrow) of the epidermis (Epi) and it is strongly attenuated in the AS (As). The expression of Mys in muscle attachments (orange star) and gut (yellow star), and the expression of DE-Cadherin (magenta) in epithelial tissues appear to be unaffected. Lateral views, anterior is left. The dotted white line highlights the epidermal edge. **(B)** Inhibition of JNK activity by overexpression of Puc (UAS-Puc) in epidermal cells (Dau-Gal4). Left, 13 AEL WT embryo and right, 15 h AEL Puc overexpression embryo. Fire Lut images represent Mys-GFP signal levels. Arrows point to the loss of Mys-GFP expression at the LE. Dorsal views. Anterior is left. See Supplementary Movies S6, S7. **(C–F)** Phase contrast dorsal images of embryonic cuticles. Anterior is up. The dorsal open phenotype of *hep* ^*1*^ mutant embryos **(C)** is partially rescued by the overexpression, in the AS cells (AS-Gal4), of a constitutively activated form of Hep (Hep^CA^) **(D)** or wild type Mys **(F)**. The dorsal hole is also partially rescued by the overexpression in the epidermis of Hep^CA^ **(E)**. (magenta stars). Scale bars are as indicated in the different panels.

This expression was modified in JNK signaling mutant embryos. In embryos lacking maternal and zygotic *hep*, Mys expression was abolished, prior to cell detachment, from the surface of the cells abutting the AS/LE interface. Moreover, Mys in AS cells was strongly reduced (Figure 2A). In contrast, the expression of Mys in muscle attachments and hindgut was not modified in *hep* mutants. This same phenotype was also observed when the inhibitory JNK phosphatase Puckered (Puc) was overexpressed in the epidermis under the control of the Daughterless-Gal4 (Dau-Gal4) line by monitoring the expression of a Mys-GFP transgene (Figure 2B and Supplementary Movies S6, S7).

Expression in the AS under the control of the *pAS-Gal4* line (compare Figures 2C,D), or in the epidermis under the control of the *Daughterless-Gal4* line (compare Figures 2C–E) of a constitutively active Hep protein (Hep^CA^) efficiently rescues the dorsal hole of *hep* mutant embryos. Partial closure was achieved in 23% of AS (*n* = 13) and 45% (*n* = 11) of epidermis Hep^CA^-expressing embryos Further, an equivalent level of rescue was achieved by overexpressing a wild type form of Mys in the AS [33% (*n* = 12)] (compare Figures 2C–F). No rescue [4% (*n* = 18) and 3% (*n* = 32)] in AS and epidermis respectively) was obtained in controls.

In both *hep* and *mys* mutant embryos, the attachment between the AS and the LE cells become partially disassembled. Altogether, these observations suggest that the JNK signaling cascade sustains the adhesion between the AS and the lateral epidermis by controlling the deployment of Mys at the leading front.

### Interference in JNK signaling and integrin expression affects the planar asymmetry of LE cells

**FIGURE 3:**
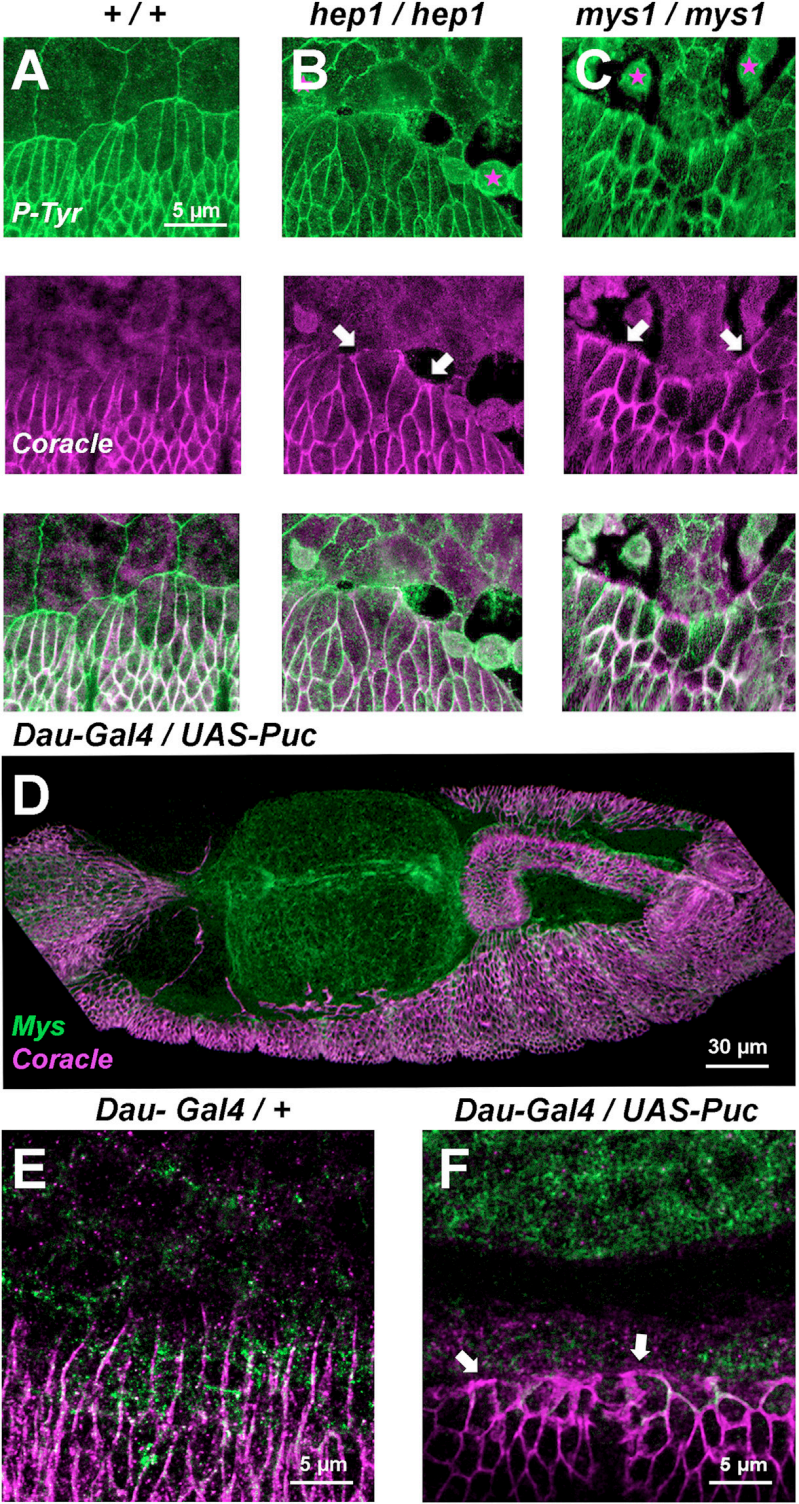
Septate junctions (Coracle) and integrins (Mys) at the LE are positioned in response to JNK signaling (**A**, **B**, and **C**) Phosphotyrosine (P-Tyr) (green) (top) and Coracle (magenta) (center) staining of stage 14 (50% completed DC) embryos (merged image - bottom). **(A)** Wild type embryo showing plasma membrane expression of P-Tyr (focal adhesions) in epidermal and AS cells. Coracle is expressed in between epidermal cells (septate junctions). LE cells show planar polarity and AS-epidermal contacts are devoid of coracle staining. In *hep* ^*1*^ **(B)** and *mys* ^*1*^ **(C)** mutant embryos, adhesion between epidermis and AS fails. LE cells lose their polarity upon detachment (arrows) and AS cells round up and eventually die. **(D)** Inhibition of JNK activity by overexpression of Puc (UAS-Puc) in epidermal cells (Dau-Gal4). Dorsolateral view of a 15 h AEL embryo. Coracle (magenta) is mislocalized and Mys expression (green) is reduced at the LE. The AS is detached from the LE. **(E)** Coracle (magenta) is asymmetrically expressed in early embryos in the absence of UAS-Puc, but carrying the Dau-Gal4 transgene. Mys expression (green) is sustained at the LE. **(F)** Inhibition of JNK activity by overexpression of Puc in epidermal cells. High magnification of a 15 h AEL embryo. Coracle (magenta) is relocated to the front of the LE and Mys expression (green) is reduced at the LE (arrows). Scale bars are as indicated in the different panels.

Coracle, a component of the septate junctions is localized at the plasma membrane of all lateral epidermis cells, except those at the leading front facing the AS, in the wild type embryo (Ward et al., 2001). Upon closure, the LE cells lose their planar asymmetry and evenly relocate Coracle (compare with the phosphotyrosine expression highlighting focal adhesions and all the epithelial cells outlines). In *hep* mutant embryos, simultaneously to the initiation of AS/LE interface disassembly, Coracle is redistributed to all surfaces of the LE cells (compare Figures 3A, B). This phenotype can also be observed in *mys* embryos when, as in *hep* mutants, LE cells detach from the AS, lose their polarity and relocate Coracle to their leading front (Figure 3C). Remarkably, when the JNK signaling cascade is blocked by the overexpression of Puc in the epidermis, LE cells also lose their planar asymmetry (Figures 3D–F). Thus, the planar asymmetry of LE cells is autonomously dependent on the activity of the JNK signaling cascade in epidermal cells and associated to their detachment from the AS.

### Mutual interactions between integrins and JNK signaling reinforce cell adhesion at the LE

**FIGURE 4:**
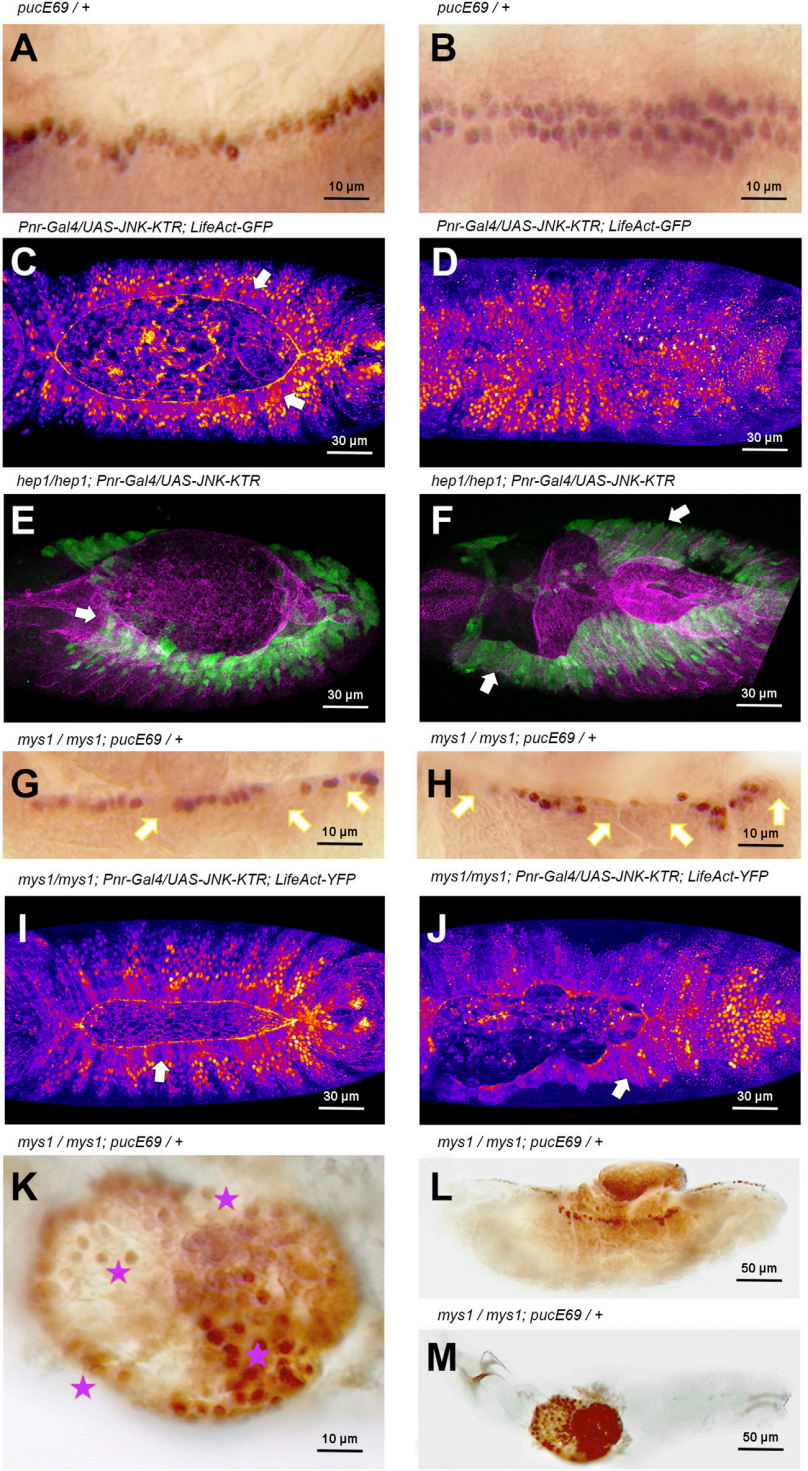
JNK signaling activity is modulated by integrin-mediated cell adhesion during DC **(A, B)** Puc-LacZ expression (anti-ß-Gal immunostaining–HRP) in LE cells during DC in wild type animals (Stage 14 and 15, 13 and 15 h AEL respectively) [**(A)**, lateral view; **(B)**, dorsal view]. Puc expression is a consequence of JNK signaling activity. Anterior is left. **(C, D)** Activity of the JNK signaling cascade at different stages [13 h AEL **(C)** and 15 h AEL **(D)**] of DC in wild type embryos, monitored by the expression of the JNK-KTR biosensor in the dorsal epidermis (Pnr-Gal4) in a genetic background carrying a *Ubi-LifeAct-YFP* transgene. LE cells, arrows in **(C)**, show cytoplasmic expression of the sensor (JNK positive activity) while the rest of the epidermis show nuclear expression. Upon closure, all epidermal cells show no JNK activity **(D)**. Fire Lut images representing KTR-GFP signal levels (white highest, dark blue lowest). Dorsal views. Anterior is left. See Supplementary Movie S8. **(E, F)** Activity of the JNK signaling cascade at different stages [13 h AEL **(E)** and 15 h AEL **(F)**] of DC in *hep1* embryos, monitored by the expression of the JNK-KTR biosensor in the dorsal epidermis (Pnr-Gal4). LE cells and lateral epidermis cells show cytoplasmic localization of the sensor (arrows) at all stages (green). Mys expression (magenta) is lost at the LE front. Dorsal views. Anterior is left. **(G, H)** Stage 14 and 15 (13 and 15 h AEL respectively) *mys* ^*1*^ mutant embryos show a decrease in the number of Puc-LacZ (anti-ß-Gal immunostaining–HRP) expressing cells at the LE (arrows) (consider β-Gal perdurance). Puc-LacZ expression is further reduced at the LE (arrows) after full detachment of the AS. Lateral view. Anterior is left. **(I, J)** Activity of the JNK signaling cascade at different stages [13 h AEL **(I)** and 15 h AEL **(J)**] of DC in *mys1* embryos in a genetic background carrying a *Ubi-LifeAct-YFP* transgene, monitored by the expression of the JNK-KTR biosensor in the dorsal epidermis. LE cells and lateral epidermis cells show cytoplasmic localization of the sensor (arrows) at all stages. Fire Lut images representing KTR-GFP signal levels (white highest, dark blue lowest). Dorsal views. Anterior is left. See Supplementary Movie S9. **(K–M))** High **(K)** and low **(L, M)** magnification of late *mys* ^*1*^ mutant embryos. A central dorsal hole is evident in the embryonic cuticle. Dying cells from internal tissues, protruding through the cuticle hole **(L)**, express high levels of Puc-LacZ (anti-ß-Gal immunostaining–HRP) (stars). Anterior is left. Scale bars are as indicated in the different panels.

During DC, the JNK pathway becomes activated early in AS and LE cells in response to unknown upstream signals. Later on, JNK activation is restricted to the LE by negative inputs in the AS mediated by Hindsight (Hnd), a nuclear Zn-finger protein, and the Puc JNK phosphatase (Reed et al., 2001), a JNK immediate early gene. *puc-LacZ* expression is detected in wild type embryos, from early DC stages, in a single continuous row of cells positioned at the LE of the lateral epidermis (Ring and Martinez Arias, 1993) (Figure 4A). It is assumed that, at the LE, the JNK pathway remains active until the end of DC when cell spreading stops, and the contralateral epidermal sheets (two rows of *puc* expressing cells) meet at the midline (Figure 4B). Yet, considering that Puc performs a direct inhibition of JNK activity, is controversial if the JNK cascade is active wherever Puc expression is detected. Thus, the JNK activation dynamics during DC has not been precisely defined. To overcome this problem, we designed a specific JNK activity fluorescent biosensor that translocates from the nuclei to the cytoplasm upon phosphorylation by JNK (JNK-KTR) (see Material and Methods) and we targeted its expression to epidermal cells in the *Drosophila* embryo. An equivalent biosensor has been employed to monitor the dynamics of JNK activity *in vivo* in human cells (Regot et al., 2014). First of all, we monitored the dynamics of JNK activity in wild type conditions and confirmed that the signaling pathway was active at the LE, during its migration towards the dorsal midline (cytoplasmic JNK-KTR expression), while JNK in the rest of the epidermis remained inactive (nuclear expression of the sensor) (Figure 4C). Upon meeting at the midline, the cells of the LE switch off JNK activity and the JNK-KTR sensor becomes nuclear (Figure 4D and Supplementary Movie S8). Then we tested the response of the sensor to the loss of function of JNK signaling and, surprisingly, we observed that in *hep* mutants, the JNK activity was sustained at the LE and it increased in the lateral epidermis (Figures 4E, F and Supplementary Movie S9). As *puc* expression strictly depends on JNK activity and since Puc negatively feedbacks on the activity of the pathway, it would be possible that in the absence of Hep, the activity of the pathway would temporarily increase. In such a scenario, the increment, rather than the pathway’s inactivity, it would be responsible for the disassembly of the AS/LE interface and the dorsal open phenotypes.

Over the past few years, it has become clear that integrins are not only involved in cell adhesion but, as well, in signal transduction. Integrins can signal directly upon stimulation by extracellular matrix proteins or could modulate growth factor-activated signaling pathways by cell anchorage to the substratum. On the other hand, integrins could trigger biochemical activation of Rac or Cdc42 GTPases, or both, in response to cell adhesion. In particular, it has been shown that Rac participates in integrin activation of JNKs in migratory cells (Cheresh et al., 1999).

These observations suggest that integrin-mediated anchorage could be involved in modulating JNK activity at the LE. We observed that in *mys* mutants, *puc*-LacZ, as a read out of JNK activity, was progressively lost from the LE cells. The loss of *puc* expression in individual cells at the leading front in mys mutants was 100% penetrant but with variable expressivity, while in WT animals no single cell losses *puc* expression. This indicates that, as a result of impaired AS/LE cells adhesion, JNK activity was downregulated (Figures 4G,H). However, as above, when employing the JNK-KTR sensor, we observed that the activity of the JNK pathway in *mys* embryos, as in *hep* mutants, was sustained at the LE and spread to the lateral epidermis (Figures 4I, J), revealing that the negative feedback loop mediating JNK activity during DC is integrin-expression dependent. Late in embryogenesis in *mys* embryos, *puc* expression was observed in extruded cells from internal organs undergoing apoptosis (Figures 4K–M). Thus, a continuous mutual crosstalk between integrins and the JNK signaling cascade affects different temporally regulated events during closure.

*In vivo* analyses employing a *puc*-Gal4 reporter fully confirmed the regulatory role of integrins on JNK activity along the different stages of DC. In *mys* mutants, *puc*-Gal4 driven GFP expression was progressively lost at the LE, as the AS detach, and at regions where the contralateral epidermis do not zip (Supplementary Figure S4 and Supplementary Movies S10, S11). Puc expression is sustained at those regions not suffering detachment suggesting that the implication of integrins in regulating JNK activity may be an indirect consequence of their function maintaining epithelial integrity.

**FIGURE 5:**
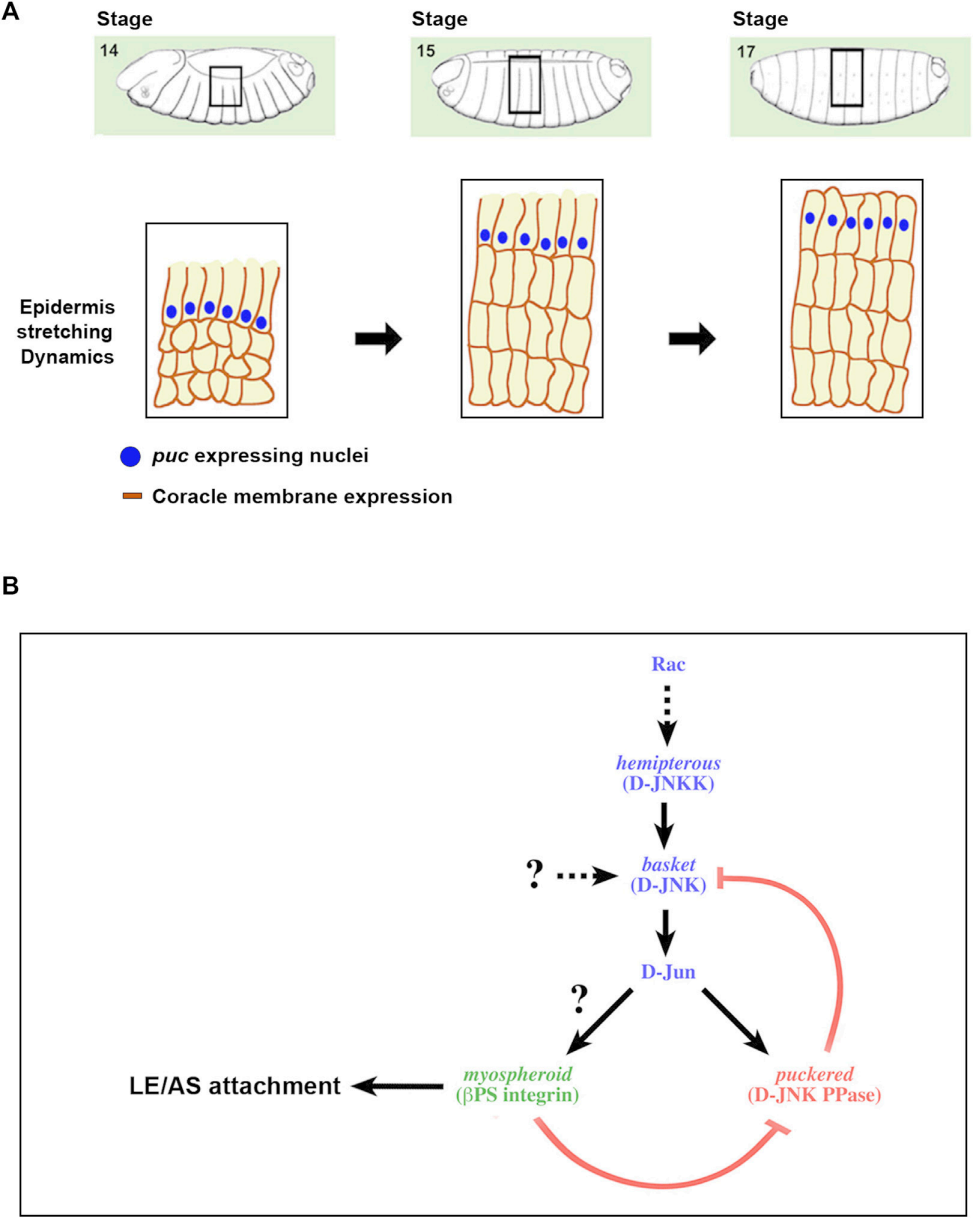
A positive feedback loop strengthening the adhesion between the epidermis and the AS during DC **(A)** Scheme depicting the different stages of DC and the description of the regulatory network linking JNK activity and integrin expression. DC initiates at Stage 13 of *Drosophila* embryogenesis with the planar polarization of the LE cells of the epidermis. Subsequently, epidermal cells coordinately stretch towards the dorsal midline, while AS cells constrict their apical ends. Finally, the embryo becomes sealed, the LE cells loss their polarity and the epidermal cells secrete the larval cuticle. Blue dots represent the nuclei of the LE cells expressing puc. Dark brown outlines of the epidermal cells’ membranes depict the expression of coracle (septate junctions), initially asymmetric and absentb of the edge confronting the AS. **(B)** As a result of the morphogenetic movements linked to DC, the epithelial sheets suffer strong mechanical tensions and sustaining tissue continuity demands a strong adhesion between the epidermal and AS cells. Alterations in the activity of the JNK signaling cascade impair these contacts, leads to AS detachment and the loss of polarity of LE cells. These processes appear to be mediated by integrins, in particular Mys, and are under the control of the JNK pathway. The pathway supported by our data is displayed. An excess of JNK activity, as an outcome of a failure in the negative feedback implemented for Puc in the epidermis, promotes the loss of Mys and AS detachment. Likewise, Mys is necessary for the regulation of JNK activity levels at the LE. In its absence, *puc* expression is down and JNK activity increases.

If integrins can activate JNK signaling, via GTPases or other ways, while JNK can promote integrin-mediated adhesion and signaling in a positive feedback loop, some interesting functional relationships become possible (Figure 5—see Discussion).

## Discussion

### The role of Cell/ECM interactions during DC

Structurally defined adhesion sites between cultured cells and the extracellular matrix (ECM) were initially described about 40 years ago. These studies revealed that matrix adhesion occurs at many specialized regions along the plasma membrane, which are tightly connected with the substrate. Amongst them, focal adhesions are associated with actin microfilaments and play an important role in cell spreading, morphogenesis and migration. The major transmembrane ECM receptors in these sites belong to the integrin family. Upon receptor clustering and occupancy in focal contacts, integrins fix cellular protrusions to the ECM, interact with the actin cytoskeleton, and trigger a hierarchic recruitment of structural and signaling molecules, such as tensin, FAK, vinculin and α-actinin. Further, ligand binding leads to local phosphorylation events [reviewed in (Keely et al., 1998; Schoenwaelder and Burridge, 1999; Revach et al., 2020)].

We and others (Wada et al., 2007) have found that the β integrin subunit Mys conspicuously accumulates at local contacts between epidermal and AS cells (Supplementary Figure S3). This expression coincides with strong, uniform phosphotyrosine staining, characteristic of focal adhesions and it is inverse to the distribution of septate junctions’ components. The leading front of the epidermis is asymmetrically polarized and devoid of septate junctions, which may favor its dynamic activity and the display of filopodia and lamellipodia.

Focal adhesions at the dorsal-most membrane of the LE are thought to generate contractile power and to act as sensors of positional information (Reed et al., 2001), while integrins on the apicolateral membrane of the AS have been suggested to serve as mechanosensors and signal specific reorganization centers for the actin (and microtubule) cytoskeleton (Meghana et al., 2011). Integrin-containing focal complexes at the AS/LE interface may respond to mechanical forces (originating from earlier morphogenetic movements such as germ band retraction) and guide directional assembly setting up the orientation of spreading during DC. In the absence of AS/LE focal complexes, the LE cells would be unable to stretch and eventually would detach (see below).

### JNK signaling activity and DC

The maternal and zygotic loss of function of *hep* leads to a lasting impairment of cell adhesion, induces the detachment of epidermal and AS cells and provoke the eventual dorsal herniation of the embryo (DC failure). Same accounts for *bsk* and other intrinsic components of the pathway as *misshapen* or *slipper* [reviewed in (Rios-Barrera and Riesgo-Escovar, 2013)]. Thus, it is assumed that JNK signaling activity is essential for closure and that its primary activity is restricted to the LE, where the immediate early gene *puc* is expressed. Indeed, the specific JNK-KTR biosensor is active at the LE and switch off in minutes immediately upon closure completion when the contralateral epidermal sheets meet at the dorsal midline (Figure 4). In *hep* and *bsk* alleles, the *puc* reporter is downregulated and dorsal closure failure has been associated to a reduction of JNK activity. Surprisingly, *puc* reappears in these mutants at late stages on herniated cells undergoing apoptosis. How this can happen? A plausible explanation is that *puc* expression in these dying cells or in cells losing cell-cell contacts could depend on the activity of non-canonical AP1 enhancers. Indeed, we recently identified a plethora of enhancers on *puc* acting in different tissues and developmental times (Llense and Martin-Blanco, 2008; Karkali and Martin-Blanco, 2021). Alternatively, the elimination, in the absence of an upstream input, of the negative feedback implemented by *puc* via dephosphorylation of JNK could, depending on the stability and/or the abundance of the phosphorylated form of the JNK, result in the net activation of the pathway. In this scenario, we found that in the absence of *hep*, the JNK-KTR biosensor remains active in the LE and it becomes ectopically active in lateral epidermal cells. This provocatively suggests that is an excess of JNK activity, and not a reduction, the causal source behind DC failure. Elevated JNK activity in the epidermis could lead to deficient cell stretching, loss of polarity at the LE, epithelial integrity defects and AS detachment.

It was already thought that focal complexes (and focal adhesions) were blocked by the inactivation of JNK signaling in the LE (Martín-Blanco et al., 1998; Reed et al., 2001). Here, we find that in *hep* mutants or after interfering with JNK signaling or eliminating *puc* in the AS and/or epidermal cells, Mys expression and actin cable assembly are disrupted and LE cells polar asymmetry is lost (Figures 2, 3). Further, LE cells change their morphology, as they appear to be released from resting tension, and both the AS and the epidermis spread away from the site of detachment (Figure 1). Cell shape changes in the AS are followed by tissue disintegration undergoing matrix detachment-induced apoptosis (anoikis) (Supplementary Figure S3). Interestingly, analyses of AS cells natural or mechanically-induced delamination show that caspase activation and apoptosis are consequences rather than the cause of their extrusion. Caspase inhibition by p35 expression does not suppress their delamination (Bahri et al., 2010). If detachment upon tissue disaggregation after interfering on JNK signaling precedes, or it is a consequence of, cells death remains to be clarified. If all these defects are associated to a net loss or a net gain on JNK activity needs to be studied further.

### A positive feedback loop between JNK signaling and integrins during DC

Studies of regulatory regions of integrin genes in vertebrates have characterized a number of integrin promoters (Kim and Yamada, 1997). Most of these promoters lack both TATA and CAAT boxes but contain Sp1-binding sites, which appear to serve as anchor sites for the TFIID initiation complex. A number of other consensus sequences have been identified in integrin promoters, including AP-1 (Fos and Jun) binding sites in several α and β subunits. Indeed, expressions of many of these genes have been shown to be responsive to known activators of Fos and Jun in cell culture. In *Drosophila*, the elaborate patterns of expression of integrins suggest the presence of complex control regions (Blair et al., 1994; D'Avino and Thummel, 2000; Fristrom and Fristrom, 1993). Likewise, several putative AP-1 sites are located 5′ upstream of the *mys* longest identified EST. Unfortunately, no promoter analysis has been performed on *mys*, or any other *Drosophila* integrin. If these sites functionally respond to JNK signaling remains to be determined. Mys was one of the genes identified in a genome-wide transcriptional search for genes responding to activation of the JNK pathway in *Drosophila* embryos (Jasper et al., 2001). Amongst several loci encoding cell adhesion molecules and cytoskeletal regulators, *mys* transcripts were enriched upon ubiquitous overexpression of Hep^CA^ along embryogenesis. Further, Mys is present throughout the embryonic epidermis and the AS and enrich at AS/LE contacts, where it responds to JNK signaling activity (Homsy et al., 2006). We confirmed these data and found that Mys expression was abolished, prior to cell detachment, from the surface of the cells abutting the AS/LE interface in embryos lacking maternal and zygotic *hep* (Figure 2). The JNK activity input in Mys expression was specific as, in this condition, it was still present in muscles attachments. Different studies have suggested integrins as key elements in the maintenance of the adhesion between the yolk membrane and the AS, the epidermis and the AS, as well as the sealing of opposing epidermis at the midline [see (Meghana et al., 2011)]. Importantly, we showed that Mys overexpression can partially rescue the dorsal open phenotype characteristic of JNK signaling mutants (Figure 2). This implies that Mys is instrumental in the maintenance of the supracellular mechanical integrity of the epidermis and the AS and critical at the AS/LE interface in response to JNK activity. JNK becomes activated at the leading front from early times. Integrins are necessary adhesion/structural factors for closure (amongst others). Importantly, they are necessary to keep a dynamic control of JNK activity. Integrins are part of the game, but the game is not played if the JNK cascade does not work.

Two additional mechanism modulating integrin-mediated adhesion in response to JNK activity could be 1) Integrin membrane recycling mediated by Rab11, a small Ras like GTPase. Cuticle preparations of embryos with altered Rab11 expression show dorsal open phenotypes. Immunohistochemical analyses of the same embryos show alterations in the localization of Mys at the AS/LE adhesion site (Bhuin and Kumar Roy, 2012) and the downregulation of *puc* expressionthe Integrin at the LE (Nandy and Roy, 2020); 2) Polarized localization of laminin A (LanA), the ECM integrin ligand at the AS/LE interface by *scarface* (*scarf*), a gene encoding for a putative secreted serine protease-like molecule without catalytic activity. Scarf expression responds to JNK signaling and *scarf* mutants show defects in DC (Sorrosal et al., 2010).

A large number of signaling molecules have been observed to be activated in response to integrin adhesion and/or clustering [reviewed in (Keely et al., 1998; Schwartz and Shattil, 2000)]. The pathway downstream of integrins could function by triggering an intracellular signaling cascade that brings about the modification of transcription factors, or alternatively, by modifying other signaling pathways. We have found that Mys is necessary for the maintenance of JNK activity at the LE of the epidermis implementing a feedback loop (Figures 4, 5). This was not fully unexpected as, in *Drosophila* S2 cells, the activation of the JNK signaling pathway by mechanical stress was integrin-dependent (Pereira et al., 2011). How could JNK activation be modulated by integrins during DC? Different mechanisms could be involved. On one hand, the LIM protein PINCH, which interacts with the Integrin-Linked Kinase (ILK) is present at areas where JNK is active and acts as a negative regulator of JNK signaling during DC (Kadrmas et al., 2004). PINCH is required for integrin-dependent adhesion and links integrins to the actin cytoskeleton. These connections could influence integrin-dependent signaling, which through a variety of tyrosine kinases and Rac would stimulate the JNK cascade. Alternatively, Rho-GEFs, known activators of GTPases and JNK activity, could be modulated by integrins through other pathways such as PI3 kinase via ILK (Wei et al., 2000) [although the *Drosophila* ILK, PI3K or Akt mutants do not show any DC defects (Reed et al., 2001; Zervas et al., 2001)]; or by Cas proteins acting as docking molecules recruiting Rho-GEFs (Dolfi et al., 1998; Gu et al., 2001).

Taken together, our results suggest that one of the main outcomes of JNK signaling during DC is to regulate the expression of Mys in the dorsal-most cells. Mys provides an effector of DC by sustaining adhesion between LE and AS cells, and also acts as a regulatory element that reinforces cell attachment. In this way, JNK initial activation might be an event stimulated by cell adhesion or cellular tension. This stimulation would in effect enhance integrin function, perhaps by inducing integrin clustering, which would promote formation of cytoskeletal or signaling assemblies. Formation of such structures would then promote a subsequent wave of integrin-dependent signals, and eventually JNK signaling reinforcement. This positive feedback mechanism provides a sensitive way of allowing the epithelial sheet to be held together against the mechanical tensions generated during closure. The self-regulation of the JNK signaling activity mediated by *puc* may convey spatio-temporal control from the LE through the different steps of the process, stretching, AS elimination, depolarization and epidermal sealing.

## Materials and methods

### *Drosophila* strains

Canton S flies were used as the wild type stock. *hep* ^*1*^ is an hypomorphic allele of the JNKK *hemipterous* (Glise et al., 1995). *mys* ^*1*^ is a zygotic null mutant of the integrin ß-subunit *myospheroid* (Glise and Noselli, 1997; Brown et al., 2000). Puckered expression (LacZ) was monitored in *pucE69/TM3 Sb* flies (Ring and Martinez Arias, 1993). A *w; UAS-Mys* ^*2.1*^ stock for Mys from N. Brown and a constitutively active Hep (Hep^CA^) (*w; UAS- Hep* ^*CA2.7*^) from S. Noselli were employed, using the UAS/Gal4 system using Pnr-Gal4 (*w; Pnr-Gal4/TM6B*) from G. Morata, pAS-Gal4 (*w; pAS-Gal4/TM3*) from S. Hayashi (Wada et al., 2007), *Daughterless-Gal4* (BDSC 27608) and *Engrailed-Gal4* (BDSC 30564). The live localization of Actin was monitored using a Moesin-GFP fusion protein under the control of a Heat-Shock promoter (*w; HS-Moe-GFP*) from D. Kiehart (Edwards et al., 1997) and a Ubi-LifeAct-YFP (*Ubi-Lifeact.YFP/CyO*) from M.D. Martin-Bermudo (Santa-Cruz Mateos et al., 2020). *Mys-GFP* was from N. Brown (Klapholz et al., 2015).

*hep* ^*1*^ homozygous females are viable producing progeny with the *hep* ^*1*^ phenotype when crossed to *hep* ^*1*^ hemizygous males. For rescue experiments using the UAS/Gal4 system, virgin females of the genotype *hep* ^*1*^ */hep* ^*1*^ *; pAS-Gal4/TM3* or *hep* ^*1*^ */hep* ^*1*^ *; Daughterless-Gal4/TM3* were crossed to males of the genotype *hep* ^*1*^ */Y; UAS-Mys* ^*2.1*^ */+* or *hep* ^*1*^ */Y; UAS- Hep* ^*CA*^ */+*.

### Live analysis

HS-Moe-GFP embryos for live analysis of actin dynamics were heat-shocked for 2 hours previous to DC, bleach dechorionated, mounted in Voltaleff oil under a coverslip and imaged live. Ubi-LifeAct-YFP was directly assessed after dechorionation and mounting. Images compiled from 20–30 confocal optical sections using Bio-Rad Radiance2000, Leica TCS SP or Zeiss LSM700 confocal systems were collected once every 5 min. The time-lapse series were assembled using Fiji software. To study DC live in mutant conditions we generated stocks carrying the HS-Moe-GFP or Ubi-LifeAct-YFP chromosomes in *hep* ^*1*^ and *mys* ^*1*^ mutant backgrounds.

### JNK activity reporter

The JNK-KTR sensor was built by subcloning KTR (Kinase Translocator Reporter) sequences (Regot et al., 2014) into a pUAST attB vector. These constructs were injected in ɸX-86Fb: y w M{eGFP.vas-int.Dm}ZH-2A; +; M{RFP.attP}ZH-86Fb; + flies to generate transgenic lines carrying Gal4 inducible reporters in the third chromosome. The JNK-KTR carries a JNK docking site, a positively phosphoregulated NES and a suboptimal bipartite NLS (bNLS) negatively regulated by phosphorylation and fused to the green fluorescent protein Clover. The negative charge introduced by the phosphorylation is responsible for the change in import and export activities of the fluorescent protein. The localization change was readily visible in different *Drosophila* tissues. Moreover, a control, nonphosphorylatable mutant (JNK-KTRAA) localized strictly to the nucleus, whereas a phosphomimetic mutant (JNK-KTREE) was restricted to the cytoplasm (data not shown).

### Immunostainings

Embryos were fixed and stained using standard protocols. Fixation was carried out using 4% paraformaldehyde diluted in PBS (phosphate-buffered saline). Specimens were incubated overnight at 4°C with primary antibodies diluted in PBT-BSA (phosphate-buffered saline containing 0.3% TritonX100% and 0.5% bovine serum albumin). The primary antibodies and concentrations used are as follows: mouse anti-Mys (βPS) integrin (1/10) (Bogaert et al., 1987); rat anti-DE-Cadherin (1/50) (Uemura et al., 1996); mouse anti-Coracle (1/100) (DSHB #C566.9); mouse anti-P-Tyr (1/400) (4G10-Upstate Biotechnology) and rabbit anti-β−Gal (1/400) (Jackson Immunoresearch). Specimens were then washed and blocked for 30 min at room temperature in PBT-BSA before a 1 h incubation with appropriate secondary antibodies (Alexa 488 and Alexa 546—fluorescent-conjugated antibodies from Molecular Probes and horseradish peroxidase (HRP) conjugated goat anti-mouse and goat anti-rabbit from Jackson Immunoresearch diluted 1:200 in PBT-BSA at room temperature.

Specimens were mounted in 50% Glycerol or Vectashield (Vector Laboratories) and examined under a Zeiss Axioplan II, a Radiance 2000 Bio-Rad and a Zeiss LSM700 confocal microscope. Superesolution was achieved in a Zeiss AiryScan LSM880 confocal microscope with a ×100 objective.

### Cuticle images

Embryonic cuticles were prepared following standard procedures (Alexandre, 2008) and monitored under Phase Contrast optics in a Zeiss Axioplan II microscope.

## Data Availability

The original contributions presented in the study are included in the article/Supplementary Material, further inquiries can be directed to the corresponding author.

## References

[B1] Affolter M. Nellen D. Nussbaumer U. Basler K. (1994). Multiple requirements for the receptor serine/threonine kinase thick veins reveal novel functions of TGF beta homologs during Drosophila embryogenesis. Development 120, 3105–3117. 10.1242/dev.120.11.3105 7720555

[B2] Alexandre C. (2008). “Cuticle preparation of Drosophila embryos and larvae,” in Drosophila: methods and protocols (Totowa, NJ: Humana Press), 197–205. 10.1007/978-1-59745-583-1_11 18641948

[B3] Bahri S. Wang S. Conder R. Choy J. Vlachos S. Dong K. (2010). The leading edge during dorsal closure as a model for epithelial plasticity: pak is required for recruitment of the Scribble complex and septate junction formation. Development 137, 2023–2032. 10.1242/dev.045088 20501591

[B4] Bhuin T. Kumar Roy J. (2012). Rab11 is required for maintenance of cell shape via βPS integrin mediated cell adhesion in Drosophila. Int. J. Mol. Cell Med. Fall 1, 185–190. PMC3920512 24551776

[B5] Blair S. S. Brower D. L. Thomas J. B. Zavortink M. (1994). The role of apterous in the control of dorsoventral compartmentalization and PS integrin gene expression in the developing wing of Drosophila. Development 120, 1805–1815. 10.1242/dev.120.7.1805 7924988

[B6] Blanchard G. B. Murugesu S. Adams R. J. Martinez-Arias A. Gorfinkiel N. (2010). Cytoskeletal dynamics and supracellular organisation of cell shape fluctuations during dorsal closure. Development 137, 2743–2752. 10.1242/dev.045872 20663818

[B7] Bogaert T. Brown N. Wilcox M. (1987). The Drosophila PS2 antigen is an invertebrate integrin that, like the fibronectin receptor, becomes localized to muscle attachments. Cell 51 (51), 929–940. 10.1016/0092-8674(87)90580-0 2961459

[B8] Brock A. R. Wang Y. Berger S. Renkawitz-Pohl R. Han V. C. Wu Y. (2012). Transcriptional regulation of Profilin during wound closure in Drosophila larvae. J. Cell Sci. 125, 5667–5676. 10.1242/jcs.107490 22976306 PMC3575702

[B9] Brown N. H. Gregory S. L. Martin-Bermudo M. D. (2000). Integrins as mediators of morphogenesis in Drosophila. Dev. Biol. 1, 1–16. 10.1006/dbio.2000.9711 10864456

[B10] Carroll J. M. Luetteke N. C. Lee D. C. Watt F. M. (1998). Role of integrins in mouse eyelid development: studies in normal embryos and embryos in which there is a failure of eyelid fusion. Mech. Dev. 78, 37–45. 10.1016/s0925-4773(98)00145-2 9858678

[B11] Cheresh D. A. Leng J. Klemke R. L. (1999). Regulation of cell contraction and membrane ruffling by distinct signals in migratory cells. J. Cell Biol. 6, 1107–1116. 10.1083/jcb.146.5.1107 PMC2169492 10477763

[B12] D'Avino P. P. Thummel C. S. (2000). The ecdysone regulatory pathway controls wing morphogenesis and integrin expression during Drosophila metamorphosis. Dev. Biol. 15, 211–224. 10.1006/dbio.2000.9650 10753511

[B13] Dolfi F. Garcia-Guzman M. Ojaniemi M. Nakamura H. Matsuda M. Vuori K. (1998). The adaptor protein Crk connects multiple cellular stimuli to the JNK signaling pathway. Proc. Natl. Acad. Sci. U. S. A. 95 (95), 15394–15399. 10.1073/pnas.95.26.15394 9860979 PMC28053

[B14] Ducuing A. Vincent S. (2016). The actin cable is dispensable in directing dorsal closure dynamics but neutralizes mechanical stress to prevent scarring in the Drosophila embryo. Nat. Cell Biol. 18, 1149–1160. 10.1038/ncb3421 27749820

[B15] Edwards K. A. Demsky M. Montague R. A. Weymouth N. Kiehart D. P. (1997). GFP-moesin illuminates actin cytoskeleton dynamics in living tissue and demonstrates cell shape changes during morphogenesis in Drosophila. Dev. Biol. 1, 103–117. 10.1006/dbio.1997.8707 9356175

[B16] Ferguson M. W. J. (1994). Craniofacial malformations: towards a molecular understanding. Nat. Genet. 6, 329–330. 10.1038/ng0494-329 7914450

[B17] Frisch S. M. Screaton R. A. (2001). Anoikis mechanisms. Curr. Opin. Cell Biol. 13, 555–562. 10.1016/s0955-0674(00)00251-9 11544023

[B18] Fristrom D. Fristrom J. W. (1993). “The metamorphic development of the adult epidermis,” in The development of Drosophila melanogaster (NewYork: Cold Spring Harbor Press), 843–898.

[B19] Glise B. Bourbon H. Noselli S. (1995). Hemipterous encodes a novel Drosophila MAP kinase kinase, required for epithelial cell sheet movement. Cell 3 (83), 451–461. 10.1016/0092-8674(95)90123-x 8521475

[B20] Glise B. Noselli S. (1997). Coupling of Jun amino-terminal kinase and Decapentaplegic signaling pathways in Drosophila morphogenesis. Genes Dev. 1 (11), 1738–1747. 10.1101/gad.11.13.1738 9224722

[B21] Goodwin K. Ellis S. J. Lostchuck E. Zulueta-Coarasa T. Fernandez-Gonzalez R. Tanentzapf G. (2016). Basal cell-extracellular matrix adhesion regulates force transmission during tissue morphogenesis. Dev. Cell 39, 611–625. 10.1016/j.devcel.2016.11.003 27923121

[B22] Gorfinkiel N. Blanchard G. B. Adams R. J. Martinez Arias A. (2009). Mechanical control of global cell behaviour during dorsal closure in Drosophila. Development 136, 1889–1898. 10.1242/dev.030866 19403661 PMC2680111

[B23] Gu J. Sumida Y. Sanzen N. Sekiguchi K. (2001). Laminin-10/11 and fibronectin differentially regulate integrin-dependent Rho and Rac activation via p130(Cas)-CrkII-DOCK180 pathway. J. Biol. Chem. 20, 27090–27097. 10.1074/jbc.M102284200 11369773

[B24] Harden N. Loh H. Y. Chia W. Lim L. (1995). A dominant inhibitory version of the small GTP-binding protein Rac disrupts cytoskeletal structures and inhibits developmental cell shape changes in Drosophila. Development 121, 903–914. 10.1242/dev.121.3.903 7720592

[B25] Homsy J. G. Jasper H. Peralta X. G. Wu H. Kiehart D. P. Bohmann D. (2006). JNK signaling coordinates integrin and actin functions during Drosophila embryogenesis. Dev. Dyn. 235, 427–434. 10.1002/dvdy.20649 16317725

[B26] Hou X. S. Goldstein E. S. Perrimon N. (1997). Drosophila Jun relays the Jun amino-terminal kinase signal transduction pathway to the Decapentaplegic signal transduction pathway in regulating epithelial cell sheet movement. Genes Dev. 1 (11), 1728–1737. 10.1101/gad.11.13.1728 9224721

[B27] Hu K. Ji L. Applegate K. T. Danuser G. Waterman-Storer C. M. (2007). Differential transmission of actin motion within focal adhesions. Science 315, 111–115. 10.1126/science.1135085 17204653

[B28] Jacinto A. Wood W. Balayo T. Turmaine M. Martinez-Arias A. Martin P. (2000). Dynamic actin-based epithelial adhesion and cell matching during Drosophila dorsal closure. Curr. Biol. 16 (10), 1420–1426. 10.1016/s0960-9822(00)00796-x 11102803

[B29] Jasper H. Benes V. Schwager C. Sauer S. Clauder-Munster S. Ansorge W. (2001). The genomic response of the Drosophila embryo to JNK signaling. Dev. Cell 1, 579–586. 10.1016/s1534-5807(01)00045-4 11703947

[B30] Kadrmas J. L. Smith M. A. Clark K. A. Pronovost S. M. Muster N. Yates J. R. 3rd (2004). The integrin effector PINCH regulates JNK activity and epithelial migration in concert with Ras suppressor 1. J. Cell Biol. 167, 1019–1024. 10.1083/jcb.200408090 15596544 PMC2034365

[B31] Karkali K. Martin-Blanco E. (2021). Dissection of the regulatory elements of the complex expression pattern of puckered, a dual-specificity JNK phosphatase. Int. J. Mol. Sci. 11, 12205. 10.3390/ijms222212205 PMC8623796 34830088

[B32] Keely P. Parise L. Juliano R. (1998). Integrins and GTPases in tumour cell growth, motility and invasion. Trends Cell Biol. 8, 101–106. 10.1016/s0962-8924(97)01219-1 9695818

[B33] Kim L. T. Yamada K. M. (1997). The regulation of expression of integrin receptors. Proc. Soc. Exp. Biol. Med. 214, 123–131. 10.3181/00379727-214-44078 9034129

[B34] Klapholz B. Herbert S. L. Wellmann J. Johnson R. Parsons M. Brown N. H. (2015). Alternative mechanisms for talin to mediate integrin function. Curr. Biol. 30 (25), 847–857. 10.1016/j.cub.2015.01.043 PMC4386027 25754646

[B35] Knust E. (1997). Drosophila morphogenesis: movements behind the edge. Curr. Biol. 7 (7), R558–R561. 10.1016/s0960-9822(06)00281-8 9285703

[B36] Lada K. Gorfinkiel N. Martinez Arias A. (2012). Interactions between the amnioserosa and the epidermis revealed by the function of the u-shaped gene. Biol. Open 1, 353–361. 10.1242/bio.2012497 23213425 PMC3509461

[B37] Laplante C. Nilson L. A. (2011). Asymmetric distribution of Echinoid defines the epidermal leading edge during Drosophila dorsal closure. J. Cell Biol. 24, 335–348. 10.1083/jcb.201009022 PMC3172166 21263031

[B38] Llense F. Martin-Blanco E. (2008). JNK signaling controls border cell cluster integrity and collective cell migration. Curr. Biol. 8 (18), 538–544. 10.1016/j.cub.2008.03.029 18394890

[B39] Lv Z. Zhang N. Zhang X. Grosshans J. Kong D. (2022). The lateral epidermis actively counteracts pulling by the amnioserosa during dorsal closure. Front. Cell Dev. Biol. 10, 865397. 10.3389/fcell.2022.865397 35652100 PMC9148979

[B40] Martin P. (1997). Wound healing--aiming for perfect skin regeneration. Science 276 (276), 75–81. 10.1126/science.276.5309.75 9082989

[B41] Martín-Blanco E. (1997). Regulation of cell differentiation by the Drosophila Jun kinase cascade. Curr. Opin. Genet. Dev. 7, 666–671. 10.1016/s0959-437x(97)80015-9 9388784

[B42] Martín-Blanco E. Gampel A. Ring J. Virdee K. Kirov N. Tolkovsky A. M. (1998). Puckered encodes a phosphatase that mediates a feedback loop regulating JNK activity during dorsal closure in Drosophila. Genes Dev. 15 (12), 557–570. 10.1101/gad.12.4.557 PMC316530 9472024

[B43] Martinez Arias A. (1993). “Development and patterning of the larval epidermis of Drosophila,” in The development of Drosophila melanogaster (NewYork: Cold Spring Harbor Press), 517–608.

[B44] Meghana C. Ramdas N. Hameed F. M. Rao M. Shivashankar G. V. Narasimha M. (2011). Integrin adhesion drives the emergent polarization of active cytoskeletal stresses to pattern cell delamination. Proc. Natl. Acad. Sci. U. S. A. 31, 9107–9112. 10.1073/pnas.1018652108 PMC3107263 21571643

[B45] Millard T. H. Martin P. (2008). Dynamic analysis of filopodial interactions during the zippering phase of Drosophila dorsal closure. Development 135, 621–626. 10.1242/dev.014001 18184725 PMC2440488

[B46] Nandy N. Roy J. K. (2020). Rab11 is essential for lgl mediated JNK-Dpp signaling in dorsal closure and epithelial morphogenesis in Drosophila. Dev. Biol. 15, 188–201. 10.1016/j.ydbio.2020.06.005 32562757

[B47] Narasimha M. Brown N. H. (2004). Novel functions for integrins in epithelial morphogenesis. Curr. Biol. 9 (14), 381–385. 10.1016/j.cub.2004.02.033 15028212

[B48] Narasimha M. Uv A. Krejci A. Brown N. H. Bray S. J. (2008). Grainy head promotes expression of septate junction proteins and influences epithelial morphogenesis. J. Cell Sci. 15 (121), 747–752. 10.1242/jcs.019422 18303052

[B49] Peralta X. G. Toyama Y. Hutson M. S. Montague R. Venakides S. Kiehart D. P. (2007). Upregulation of forces and morphogenic asymmetries in dorsal closure during Drosophila development. Biophys. J. 1 (92), 2583–2596. 10.1529/biophysj.106.094110 PMC1864829 17218455

[B50] Pereira A. M. Tudor C. Kanger J. S. Subramaniam V. Martin-Blanco E. (2011). Integrin-dependent activation of the JNK signaling pathway by mechanical stress. PLoS One 6, e26182. 10.1371/journal.pone.0026182 22180774 PMC3236745

[B51] Raich W. B. Agbunag C. Hardin J. (1999). Rapid epithelial-sheet sealing in the *Caenorhabditis elegans* embryo requires cadherin-dependent filopodial priming. Curr. Biol. 9, 1139–1146. 10.1016/S0960-9822(00)80015-9 10531027

[B52] Reed B. H. Wilk R. Lipshitz H. D. (2001). Downregulation of Jun kinase signaling in the amnioserosa is essential for dorsal closure of the Drosophila embryo. Curr. Biol. 24 (11), 1098–1108. 10.1016/s0960-9822(01)00318-9 11509232

[B53] Reed B. H. Wilk R. Schöck F. Lipshitz H. D. (2004). Integrin-dependent apposition of Drosophila extraembryonic membranes promotes morphogenesis and prevents anoikis. Curr. Biol. 9 (14), 372–380. 10.1016/j.cub.2004.02.029 15028211

[B54] Regot S. Hughey J. J. Bajar B. T. Carrasco S. Covert M. W. (2014). High-sensitivity measurements of multiple kinase activities in live single cells. Cell 157, 1724–1734. 10.1016/j.cell.2014.04.039 24949979 PMC4097317

[B55] Revach O.-Y. Grosheva I. Geiger B. (2020). Biomechanical regulation of focal adhesion and invadopodia formation. J. Cell Sci. 133, jcs244848. 10.1242/jcs.244848 33093229

[B56] Riesgo-Escovar J. R. Hafen E. (1997). Drosophila Jun kinase regulates expression of decapentaplegic via the ETS-domain protein Aop and the AP-1 transcription factor DJun during dorsal closure. Genes Dev. 11 (11), 1717–1727. 10.1101/gad.11.13.1717 9224720

[B57] Ring J. M. Martinez Arias A. (1993). puckered, a gene involved in position-specific cell differentiation in the dorsal epidermis of the Drosophila larva. Dev. Suppl. 119, 251–259. 10.1242/dev.119.supplement.251 8049480

[B58] Rios-Barrera L. D. Riesgo-Escovar J. R. (2013). Regulating cell morphogenesis: the Drosophila Jun N-terminal kinase pathway. Genesis 51, 147–162. 10.1002/dvg.22354 23109363

[B59] Rugendorff A. Younossi-Hartenstein A. Hartenstein V. (1994). Embryonic origin and differentiation of the Drosophila heart. Rouxs Arch. Dev. Biol. 203, 266–280. 10.1007/BF00360522 28305624

[B60] Santa-Cruz Mateos C. Valencia-Exposito A. Palacios I. M. Martin-Bermudo M. D. (2020). Integrins regulate epithelial cell shape by controlling the architecture and mechanical properties of basal actomyosin networks. PLoS Genet. 16, e1008717. 10.1371/journal.pgen.1008717 32479493 PMC7263567

[B61] Schoenwaelder S. M. Burridge K. (1999). Bidirectional signaling between the cytoskeleton and integrins. Curr. Opin. Cell Biol. 11, 274–286. 10.1016/s0955-0674(99)80037-4 10209151

[B62] Schwartz M. A. Shattil S. J. (2000). Signaling networks linking integrins and rho family GTPases. Trends Biochem. Sci. 25, 388–391. 10.1016/s0968-0004(00)01605-4 10916159

[B63] Solon J. Kaya-Copur A. Colombelli J. Brunner D. (2009). Pulsed forces timed by a ratchet-like mechanism drive directed tissue movement during dorsal closure. Cell 137 (137), 1331–1342. 10.1016/j.cell.2009.03.050 19563762

[B64] Sorrosal G. Pérez L. Herranz H. Milán M. (2010). Scarface, a secreted serine protease-like protein, regulates polarized localization of laminin A at the basement membrane of the Drosophila embryo. EMBO Rep. 11, 373–379. 10.1038/embor.2010.43 20379222 PMC2868543

[B65] Stevens L. J. Page-McCaw A. (2012). A secreted MMP is required for reepithelialization during wound healing. Mol. Biol. Cell 23, 1068–1079. 10.1091/mbc.E11-09-0745 22262460 PMC3302734

[B66] Toyama Y. Peralta X. G. Wells A. R. Kiehart D. P. Edwards G. S. (2008). Apoptotic force and tissue dynamics during Drosophila embryogenesis. Science 19, 1683–1686. 10.1126/science.1157052 PMC2757114 18802000

[B67] Uemura T. Oda H. Kraut R. Hayashi S. Kotaoka Y. Takeichi M. (1996). Zygotic Drosophila E-cadherin expression is required for processes of dynamic epithelial cell rearrangement in the Drosophila embryo. Genes Dev. 15 (10), 659–671. 10.1101/gad.10.6.659 8598294

[B68] Wada A. Kato K. Uwo M. F. Yonemura S. Hayashi S. (2007). Specialized extraembryonic cells connect embryonic and extraembryonic epidermis in response to Dpp during dorsal closure in Drosophila. Dev. Biol. 301, 340–349. 10.1016/j.ydbio.2006.09.020 17034783

[B69] Wang M. C. Bohmann D. Jasper H. (2003). JNK signaling confers tolerance to oxidative stress and extends lifespan in Drosophila. Dev. Cell 5, 811–816. 10.1016/s1534-5807(03)00323-x 14602080

[B70] Ward R. Schweizer L. Lamb R. S. Fehon R. G. (2001). The protein 4.1, ezrin, radixin, moesin (FERM) domain of Drosophila Coracle, a cytoplasmic component of the septate junction, provides functions essential for embryonic development and imaginal cell proliferation. Genetics 159, 219–228. 10.1093/genetics/159.1.219 11560899 PMC1461787

[B71] Wei L. Zhou W. Wang L. Schwartz R. J. (2000). beta(1)-integrin and PI 3-kinase regulate RhoA-dependent activation of skeletal alpha-actin promoter in myoblasts. Am. J. Physiol. Heart Circ. Physiol. 278, H1736–H1743. 10.1152/ajpheart.2000.278.6.H1736 10843867

[B72] Wright T. R. (1960). The phenogenetics of the embryonic mutant, lethal myospheroid, in *Drosophila melanogaster* . J. Exp. Zool. 143, 77–99. 10.1002/jez.1401430107 13786827

[B73] Young P. E. Richman A. M. Ketchum A. S. Kiehart D. P. (1993). Morphogenesis in Drosophila requires nonmuscle myosin heavy chain function. Genes Dev. 7, 29–41. 10.1101/gad.7.1.29 8422986

[B74] Zeitlinger J. Kockel L. Peverali F. A. Jackson D. B. Mlodzik M. Bohmann D. (1997). Defective dorsal closure and loss of epidermal decapentaplegic expression in Drosophila fos mutants. Embo J. 16 (16), 7393–7401. 10.1093/emboj/16.24.7393 9405368 PMC1170339

[B75] Zervas C. G. Gregory S. L. Brown N. H. (2001). Drosophila integrin-linked kinase is required at sites of integrin adhesion to link the cytoskeleton to the plasma membrane. J. Cell Biol. 5 (152), 1007–1018. 10.1083/jcb.152.5.1007 PMC2198807 11238456

