# Supplementary figures and images for: JNK signaling and integrins cooperate to maintain cell adhesion during epithelial fusion in Drosophila
Source: Front Cell Dev Biol. 2024 Jan 9;11:1034484. doi: 10.3389/fcell.2023.1034484 (PMC10803605; doi:10.3389/fcell.2023.1034484)

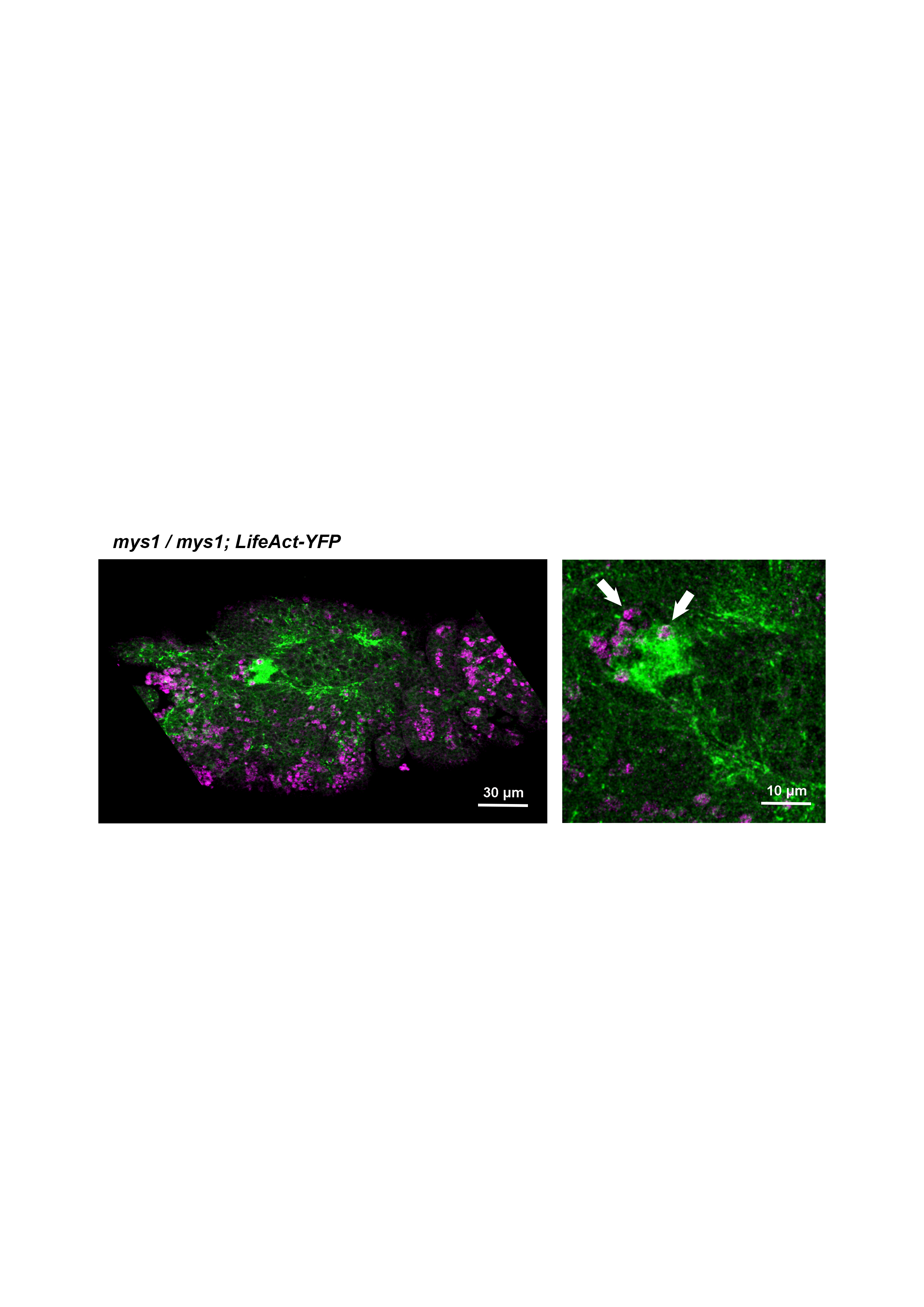

Supplement: Supplementary file 4 [file Image3.TIF]

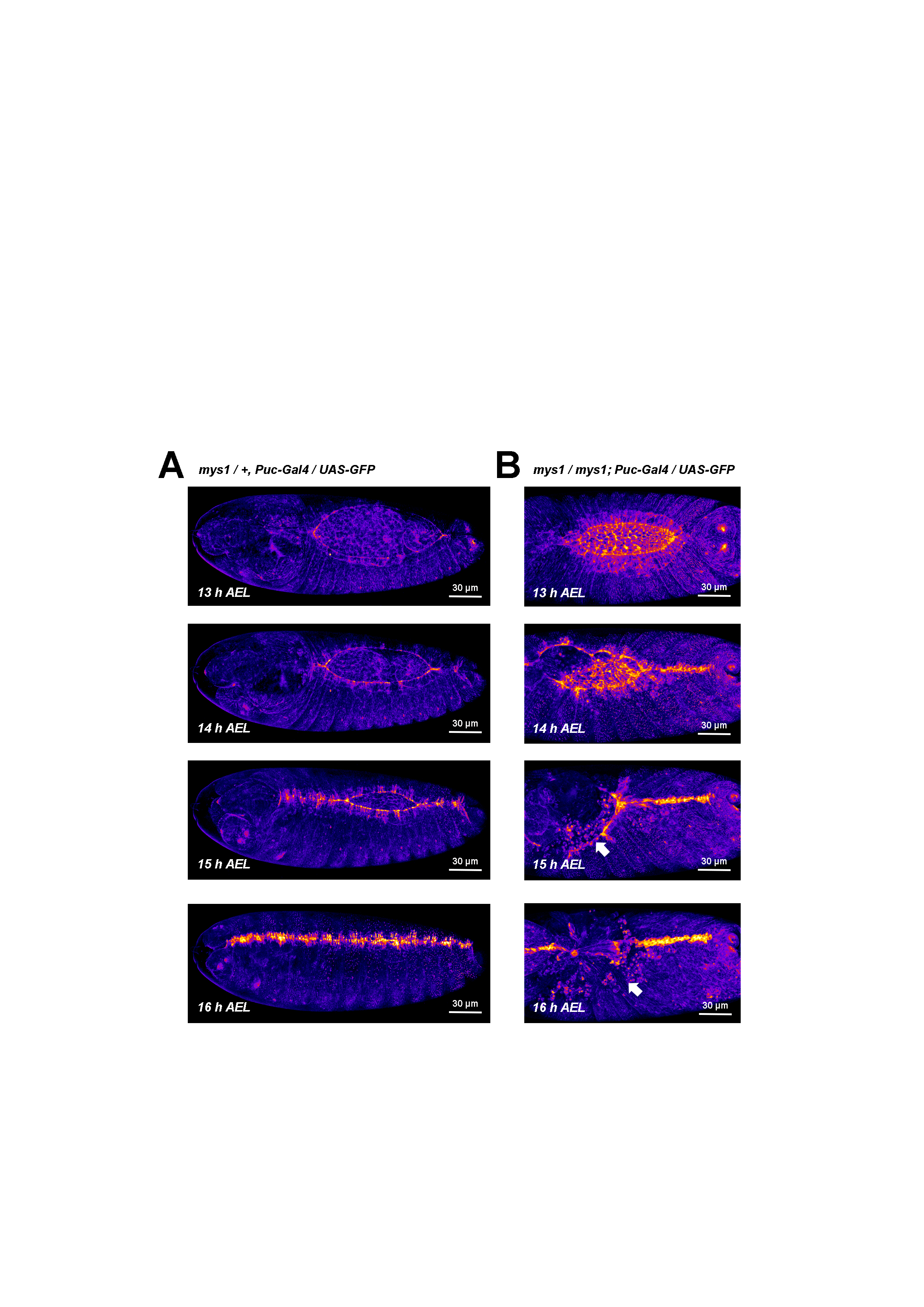

Supplement: Supplementary file 5 [file Image4.TIF]

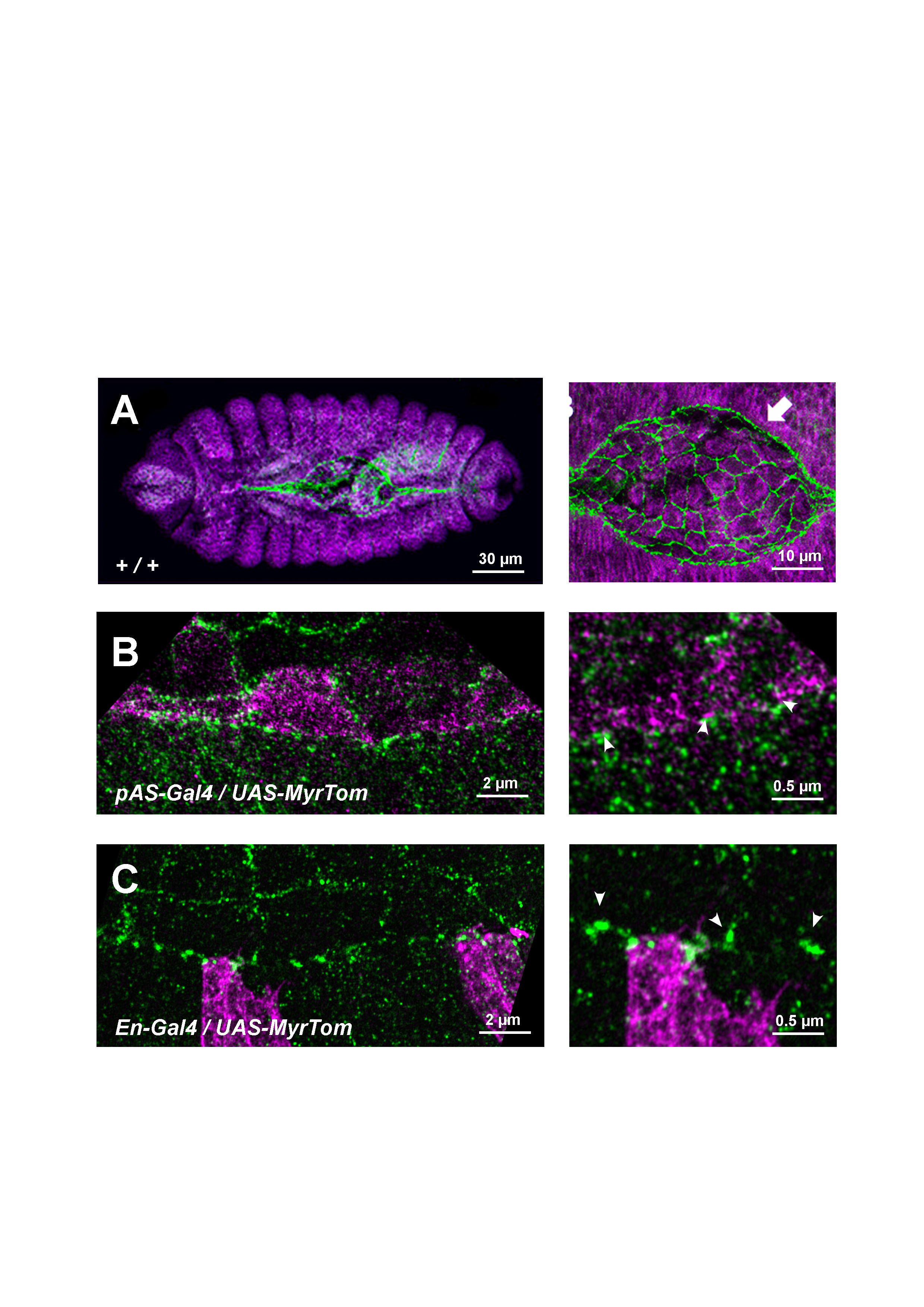

Supplement: Supplementary file 6 [file Image2.TIF]

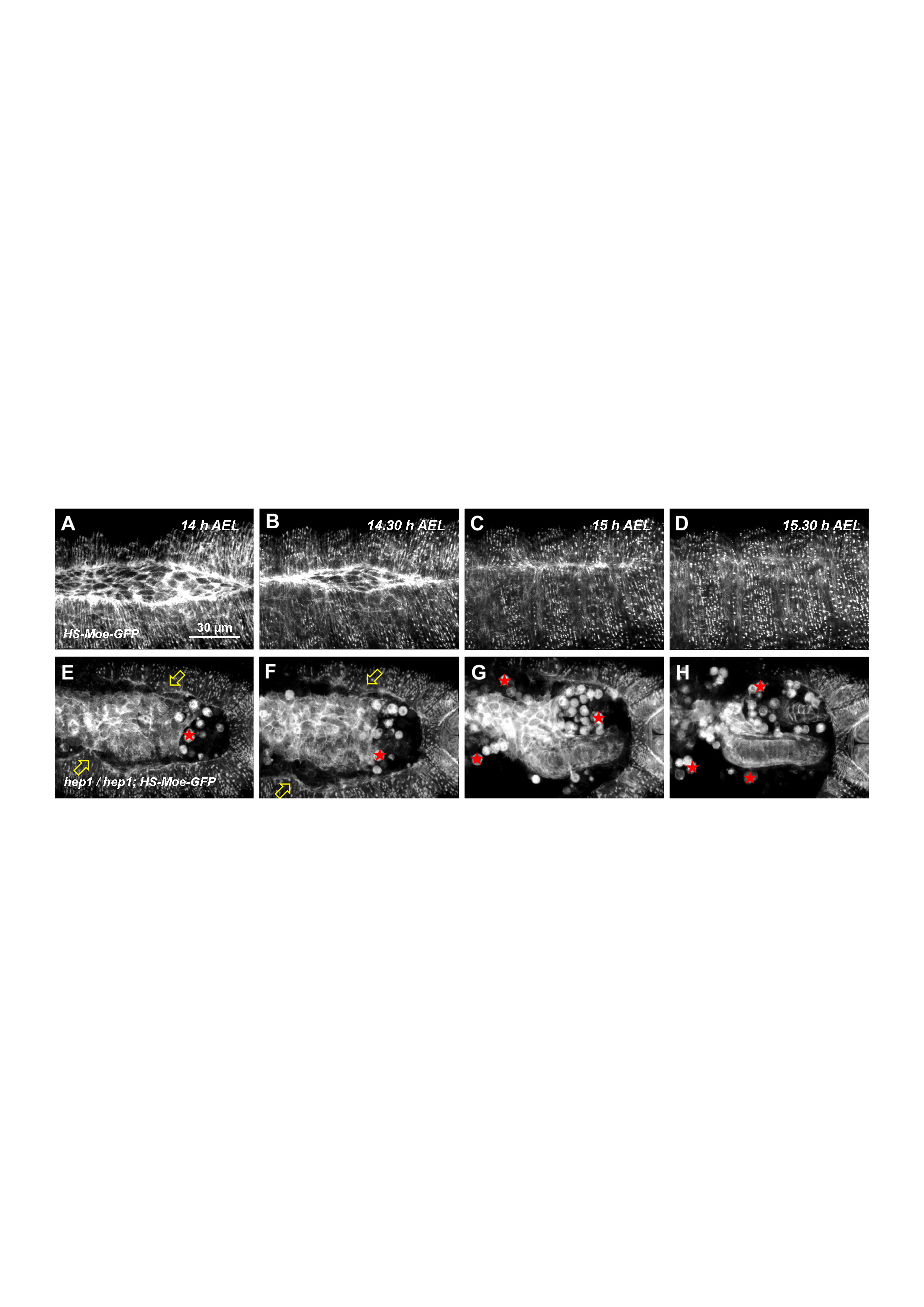

Supplement: Supplementary file 7 [file Image1.TIF]
