# Supplementary material for: JNK signaling and integrins cooperate to maintain cell adhesion during epithelial fusion in Drosophila
Source: Front Cell Dev Biol. 2024 Jan 9;11:1034484. doi: 10.3389/fcell.2023.1034484 (PMC10803605; doi:10.3389/fcell.2023.1034484)
Supplement: Supplementary file 8 [file DataSheet1.pdf]

JNK signaling and integrins cooperate to maintain cell adhesion during epithelial fusion in *Drosophila*

Katerina Karkali, José Carlos Pastor-Pareja and Enrique Martín-Blanco

SUPPLEMENTAL MATERIAL

Supplemental Figure 1. Low magnification micrographs of wild type and *hep* mutant embryos through DC

Embryos with dorsal orientations give an overall view of the adhesion defects observed in JNK signaling mutants (HS-Moe-GFP - see Materials and Methods). **(A, E)** DC is 40% complete. **(B, F)** DC is 75% complete. **(C, G)** DC is completed. **(D, H**) Stage 17 embryos. In wild type embryos, the LE of the lateral epidermis begins to accumulate actin **(A)** and cells change shape from polygonal to elongate. Further, AS cells show dramatically constricted apical sides **(B)**. Later on, the lateral epidermis is fully extended and the contralateral LEs meet at the midline **(C)**. Last, the embryonic cuticle is entirely deployed and no scar is observed **(D)**. In *hep*^{1} mutants, the epidermis becomes detached from the AS and actin is lost from the LE **(E)**. Afterwards **(F)**, the epidermis has fully detached from the AS and initiates autonomous retraction (arrows). The AS cells undergo detachment-induced cell death (anoikis) (stars). Thus, the AS is virtually disassembled **(G)** and the gut and other internal tissues protrude out through the dorsal hole left by the retracted epidermis **(H)**. See Supplemental Movies 2 and 4.

Supplemental Figure 2. Colocalization of AS and epidermal markers with Mys

A) Stage 14 wild type embryo. Mys (green) is expressed at high levels in AS cells and at the leading front of LE cells (arrow). DE-Cadherin is expressed in both epidermal and AS cells (magenta). Dorsal views, anterior is left.

B) Colocalization of Mys (green) and AS membrane markers (pAS-Gal4 / UAS-Myr-Tomato) (magenta). Superesolution analysis highlighting a double row (arrowheads) of Mys puncta at the AS/LE interface.

C) Colocalization of Mys (green) and epidermal cells membrane markers (En-Gal4 / UAS-Myr-Tomato) (magenta). Superesolution analysis highlighting a double row (arrowheads) of Mys puncta at the AS/LE interface.

Supplemental Figure 3. Cell death in *mys* mutant embryos

Generalized cell death detected by the expression of Dcp1 (magenta) in *mys1* mutant embryos. The actin cytoskeleton (green) is deranged and ectopic bundles expressing LifeAct were observed.

Supplemental Figure 4.

A) Dynamics of *puc* expression (Puc-Gal4/UAS-GFP) in wild type heterozygous *mys1* animals. *puc* expression initiates at 13 h AEL at the LE and is restricted to the front row of the epidermal cells. These cells meet at the dorsal midline by 16 h AEL. See Supplemental Movie 10.

B) In *mys1* homozygous embryos *puc* expression is kept restricted to the LE but gets progressively lost at those positions where the AS detaches from the epidermis (arrows). Puc expression is sustained in those contralateral domains where detachment does not occur and where the epidermis succeeds to meet at the dorsal midline. See Supplemental Movie 11.

Movie 1. LifeAct-YFP – Dorsal view of a wild type embryo

Movie of a *w/FM7; Ubi-LifeAct-YFP* embryo imaged using time-lapse confocal microscopy showing late stages of DC. A thick actin cable is form at the LE cells. The epithelia move forward and the AS invaginates. The contralateral epidermal sheets meet at the dorsal midline. Anterior is left.

Movie 2. Moe-GFP - Dorsal view of wild type embryo

Movie of a *HS-Moe-GFP/+* embryo imaged using time-lapse confocal microscopy showing late stages of DC. A thick actin cable is form at the LE cells. The epithelia move forward and the AS invaginates. Finally, the epidermis secretes the larval cuticle and no scar is left. Note the segmental iteration of epithelial fusion. Anterior is left.

Movie 3. LifeAct-YFP – Dorsal view of a *hep1* mutant embryo

Movie of a *hep*^{1}/*hep*^{1}; *Ubi-LifeAct-YFP* embryo with no maternal contribution imaged using time-lapse confocal microscopy showing late stages of DC. The epithelial cells detach at multiple positions from the AS and this disassembles. Anterior is left.

Movie 4. Moe-GFP – Dorsal view of a *hep*^{1} mutant embryo

Movie of a *hep*^{1}/*hep*^{1}; *HS-Moe-GFP/+* embryo imaged using time-lapse confocal microscopy showing late stages of DC. The epithelial cells progressively detach from the AS. Later on, AS cells detach from each other and undergo apoptosis. Finally, the internal structures protrude out through the massive dorsal hole. Anterior is left.

Movie 5. LifeAct-YFP – Dorsal view of a *mys1* mutant embryo

Movie of a *mys*^{1}/*mys*^{1}; *Ubi-LifeAct-YFP* embryo. As a result of the absence of *mys*, the AS and the epidermis detach a dorsal hole is created anteriorly. The gut and other internal structures are extruded. Note the retraction of both epidermis and AS as a consequence of the loss of continuity of the epithelia at the LE. Anterior is left.

Movie 6. Mys-GFP expression in a wild type embryo – Dorsal view

Movie of a *Mys-GFP; Dau-Gal4/+* embryo imaged using time-lapse confocal microscopy showing late stages of DC. Mys is initially expressed in the AS and at the LE. This expression is sustained until the end of closure. Later on, strong expression is built up at the transversal muscle attachments iterated in all segments. Expression intensity is emphasized by a Fire Lut. Anterior is left.

Movie 7. Mys-GFP expression in an embryo expressing Puc in the epidermis – Dorsal view

Movie of a *Mys-GFP; Dau-Gal4/UAS-Puc* embryo imaged using time-lapse confocal microscopy showing late stages of DC. The expression of Puc in the epidermis driven by the Dau-Gal4 results in the detachment of the AS from the LE and dorsal holes are created. Mys expression is lost from the LE as it detaches from the AS. Expression in the muscles attachments is sustained. Expression intensity is emphasized by a Fire Lut. Anterior is left.

Movie 8. Expression dynamics of the JNK-KTR biosensor expressed in the dorsal epidermis – dorsal view

Movie of a *Pnr-Gal4/UAS-JNK-KTR; Ubi-LifeAct-YFP* embryo imaged using time-lapse confocal microscopy showing late stages of DC. The expression of the JNK-KTR sensor in the dorsal epidermis driven by the Pnr-Gal4 shows cytoplasmic expression (JNK activity) at the LE, while the rest of the epidermis shows nuclear expression (inactive JNK). Upon closure, all cells switch of JNK activity and the nuclear staining is uniform. LifeAct highlights the AS and the LE front actin cable. Expression intensity is emphasized by a Fire Lut. Anterior is left.

Movie 9. Expression dynamics of the JNK-KTR biosensor expressed in the dorsal epidermis of a *mys1* mutant embryo – dorsal view

Movie of a *mys1/mys1; Pnr-Gal4/UAS-JNK-KTR; Ubi-LifeAct-YFP* embryo imaged using time-lapse confocal microscopy showing late stages of DC. The expression of the JNK-KTR

sensor in the dorsal epidermis driven by the Pnr-Gal4 initially shows cytoplasmic expression (JNK activity) at the LE, while the rest of the epidermis shows nuclear expression (inactive JNK). As the AS detaches from the LE, all epidermal cells activate JNK (cytoplasmic expression). At late stages JNK activity is switched off. LifeAct highlights the AS and the LE front actin cable. Expression intensity is emphasized by a Fire Lut. Anterior is left.

Movie 10. Expression dynamics of Puc during DC – dorsal view

Movie of a *mys1/+; Puc-Gal4/UAS-GFP* embryo imaged using time-lapse confocal microscopy showing late stages of DC. The expression of *puc* initiates at the LE as DC starts. The expression increased overtime and it is sustained after closure is accomplished. Expression intensity is emphasized by a Fire Lut. Anterior is left.

Movie 11. Expression dynamics of Puc during DC in *mys1* mutants – dorsal view

Movie of a *mys1/mys1; Puc-Gal4/UAS-GFP; LifeAct-YFP* embryo imaged using time-lapse confocal microscopy showing late stages of DC. The expression of *puc* initiates at the LE as DC starts. The expression of *puc* is slowly lost from the LE cells as they detach from the AS (consider the slow decay of the GFP signal). Puc expression is still observed in those areas where closure succeed and the contralateral epidermal sheets meet at the midline. LifeAct highlights the AS and the LE front actin cable. Expression intensity is emphasized by a Fire Lut. Anterior is left.
